# Acceptability, effectiveness and cost-effectiveness of blended cognitive-behavioural therapy (bCBT) versus face-to-face CBT (ftfCBT) for anxiety disorders in specialised mental health care: A 15-week randomised controlled trial with 1-year follow-up

**DOI:** 10.1371/journal.pone.0259493

**Published:** 2021-11-12

**Authors:** Geke Romijn, Neeltje Batelaan, Jeroen Koning, Anton van Balkom, Aart de Leeuw, Friederike Benning, Leona Hakkaart van Roijen, Heleen Riper

**Affiliations:** 1 Clinical Psychology Section, Department of Clinical, Neuro- and Developmental Psychology, Vrije Universiteit Amsterdam; and Amsterdam Public Health Research Institute, Amsterdam, The Netherlands; 2 Specialised Mental Health Institution, GGz Breburg, Tilburg, the Netherlands; 3 Altrecht Academic Anxiety Centre, Utrecht, Netherlands; 4 Amsterdam UMC, Vrije Universiteit Amsterdam, Department of Psychiatry, Amsterdam Public Health Research Institute and GGZ inGeest Specialized Mental Health Care, Amsterdam, The Netherlands; 5 Institute for Psychiatry, Vincent van Gogh, Venray, The Netherlands; 6 Department of Health Technology Assessment, Erasmus School of Health Policy and Management, Rotterdam, The Netherlands; 7 Centre for Telepsychiatry, Mental Health Services of Southern Denmark, Odense, Denmark; Brown University, UNITED STATES

## Abstract

**Background:**

Anxiety disorders are highly prevalent and cause substantial economic burden. Blended cognitive-behavioural therapy (bCBT), which integrates Internet-based CBT and face-to-face CBT (ftfCBT), is an attractive and potentially cost-saving treatment alternative to conventional CBT for patients with anxiety disorders in specialised mental health care. However, little is known about the effectiveness of bCBT in routine care. We examined the acceptability, effectiveness and cost-effectiveness of bCBT versus ftfCBT in outpatient specialised care to patients with panic disorder, social anxiety disorder and generalised anxiety disorder.

**Methods and findings:**

Patients with anxiety disorders were randomised to bCBT (*n* = 52) or ftfCBT (*n* = 62). Acceptability of bCBT and ftfCBT were evaluated by assessing treatment preference, adherence, satisfaction and therapeutic alliance. Costs and effects were assessed at post-treatment and one-year follow-up. Primary outcome measure was the Beck Anxiety Inventory (BAI). Secondary outcomes were depressive symptoms, general psychopathology, work and social adjustment, quality of life and mastery. Incremental cost-effectiveness ratios (ICERs) were computed from societal and healthcare perspectives by calculating the incremental costs per incremental quality-adjusted life year (QALY). No significant differences between bCBT and ftfCBT were found on acceptability or effectiveness measures at post-treatment (Cohen’s *d* between-group effect size on BAI = 0.15, 95% CI −0.30 to 0.60) or at one-year follow-up (*d* = −0.38, 95% CI −0.84 to 0.09). The modelled point estimates of societal costs (bCBT €10945, ftfCBT €10937) were higher and modelled point estimates of direct medical costs (bCBT €3748, ftfCBT €3841) were lower in bCBT. The acceptability curves showed that bCBT was expected to be a cost-effective intervention. Results should be carefully interpreted due to the small sample size.

**Conclusions:**

bCBT appears an acceptable, clinically effective and potentially cost-saving alternative option for treating patients with anxiety disorders. Trials with larger samples are needed to further investigate cost-effectiveness.

**Trial registration:**

Netherlands Trial Register: NTR4912.

## Background

Anxiety disorders are highly prevalent, and they are associated with considerable individual suffering and a high economic burden [1–4]. The disorders can be treated effectively with cognitive-behavioural therapy (CBT) [5, 6]. Despite the demonstrated effectiveness of CBT, fewer than half of the people with anxiety disorders receive appropriate treatment [7]. Reasons for undertreatment include stigmatisation, lack of trained therapists and the costs of therapy [8].

One strategy to expand access to evidence-based therapy while lowering treatment costs could be Internet-delivered CBT (iCBT). Patients receiving iCBT usually work their way through an online modularised programme, with or without online therapist assistance [9]. iCBT has been found effective for several anxiety disorders [10–13], and there are indications for its cost-effectiveness [14]. However, most evidence thus far derives from research outside routine clinical care settings. It has not been established that the promising results from those effectiveness studies can be extrapolated to samples in routine care. For example, in a recent meta-analysis [12], a large effect size (*g* = 0.79) was found for anxiety symptom reduction by iCBT as compared with waitlisted controls in samples recruited from the community, while a small effect size (*g* = 0.28) was found in the same comparison in routine care populations. A possible explanation for the discrepancy was the greater treatment adherence in self-referred samples recruited from the community and the stricter exclusion criteria in studies with such samples.

The low uptake of iCBT in routine care complicates the investigation of effectiveness in real-world settings. Reported reasons for therapists’ reluctance to use iCBT are their concerns about the therapeutic relationship [15] and low treatment adherence, especially in patients with high symptom severity [16, 17]. Blended CBT (bCBT) combines iCBT and ftfCBT into a single standardised treatment protocol [18] and could potentially alleviate some of the aforementioned limitations associated with iCBT, while partly or fully preserving the advantages. It could help provide an attractive, and potentially cost-saving, treatment alternative for use in conventional mental health care settings. For one thing, bCBT has been found to be better received by both providers and patients than iCBT, because the face-to-face contact in the blended format makes the treatment more personal, better addresses the needs of patients with complex symptomatology, and may help improve adherence rates [15, 19–21]. A further possible advantage is that online components can be integrated into routine practice more gradually [22], making the blended format easier than iCBT to adopt for application in routine care.

Although bCBT thus seems a promising alternative to both iCBT and ftfCBT, little is known so far about the clinical and cost benefits of blended interventions for anxiety disorders. In a feasibility randomised controlled trial (RCT) comparing bCBT (*n* = 18) with ftfCBT (*n* = 18) for panic disorder, no difference was found between bCBT and ftfCBT in reducing anxiety symptoms [23].

As bCBT could possibly reduce therapist time [24] and improve self-management competencies of patients in comparison with ftfCBT, providing bCBT to patients with severe anxiety disorders in specialised mental health care might lead to equal clinical effectiveness results at lower treatment costs. We thus hypothesised that bCBT is more cost-effective than ftfCBT. We undertook a randomised controlled trial to investigate the acceptability and the clinical and cost-effectiveness of bCBT for patients with panic disorder (PD), social anxiety disorder (SAD) and generalised anxiety disorder (GAD) in outpatient specialised mental health care. The current paper describes the acceptability, the post-treatment and 12-month clinical effectiveness, and the 12-month cost-effectiveness of bCBT versus ftfCBT from both a societal and a healthcare perspective.

## Methods

### Study design and participants

The study design was a parallel-group randomised controlled trial. The purpose was to assess acceptability, effectiveness and cost-effectiveness of bCBT compared with ftfCBT in patients with panic disorder, social phobia or generalised anxiety disorder in routine specialised mental health care. Assessments took place at post-treatment and at one-year follow-up, respectively 15 and 52 weeks after baseline. Patients who are referred to specialized mental health care in the Netherlands are suffering from serious mental disorders [25]. Hence, participants were likely to have received psychological treatment within primary care before they were enrolled in this trial. Patients in both treatment conditions were allowed to receive other supporting therapy after the intervention.

Participant inclusion criteria were (i) age 18 or older and (ii) satisfaction of the DSM-IV criteria for panic disorder (with or without agoraphobia), social anxiety disorder or generalised anxiety disorder, as diagnosed with the Structured Clinical Interview for DSM-IV Axis I Disorders (SCID-I) [26], or the Mini-International Neuropsychiatric Interview, Plus version (MINI-Plus) [27, 28]. Exclusion criteria were (i) inadequate proficiency in Dutch, (ii) lack of e-mail address or computer with Internet access and (iii) presence of a psychotic or bipolar disorder, substance dependence or a high risk for suicide. Psychotropic medication use was allowed.

A detailed study protocol has been published elsewhere [29]. The protocol was approved by the Medical Ethics Committee of the Vrije Universiteit Medical Centre, Amsterdam (registration number 2015.073), and registered in the Netherlands Trial Register (NTR4912). The study protocol and supporting CONSORT checklist and CHEERS checklist for this trial are available as supporting information; see S1 Appendix (CONSORT Checklist), S2 Appendix (CHEERS Checlist) and S3 Appendix (Study Protocol).

### Recruitment

Recruitment took place between November 2015 and July 2017 at outpatient departments of four specialised mental health care centres in the Netherlands. Mental health professionals who conducted the therapy intake session requested feasible patients’ permission to be contacted by one of the researchers. The researcher briefed interested patients about the study, sent them all relevant information on the trial, and invited them for the baseline diagnostic interview (face-to-face or by telephone). For study inclusion, the primary diagnosis was to be confirmed in that interview by a research assistant using the MINI-Plus or SCID-I. Any comorbid DSM-IV diagnoses were also assessed with the MINI-Plus or SCID-I. Written informed consent was obtained from all participants before baseline assessment and randomisation.

### Randomisation

After the baseline assessment, the included participants were randomly allocated to either bCBT or ftfCBT by an independent researcher using a computer-generated block randomisation table. Randomisation was stratified across the four research sites. Due to the nature of the intervention, patients and therapists could not be blinded to treatment allocation.

### Interventions

Cognitive-behavioural therapy (CBT) was provided in both treatment conditions, including evidence-based components for treatment of anxiety disorders: psychoeducation, cognitive therapy, exposure and relapse prevention [5, 6, 25]. The treatment protocols were based on the standard Dutch treatment protocols [30]. For the blended variants, face-to-face and online sessions were integrated into a single blended treatment protocol for each disorder.

Therapists taking part in the study delivered therapy to patients in both treatment conditions. All therapists had formal training and experience in delivering CBT and had received training in the delivery of the blended format.

For the three primary diagnoses, three different treatment protocols were used. In the event of comorbid anxiety disorders, the protocol of choice was based on the patient’s most prominent disorder, as established during the therapy intake session. The treatment sessions contained psychoeducation (explanation of treatment rationale and general procedures in cognitive therapy), cognitive therapy (examination of relationships between thoughts, emotions and behaviour), exposure tasks (graded exposure to feared situations) and relapse prevention (identification and adoption of strategies to prevent anxiety symptoms from reoccurring). Cognitive therapy for PD and SAD focused on reinterpreting the causes and consequences of anxiety symptoms. The protocol for GAD consisted of metacognitive therapy, which identifies underlying metabeliefs about worrying and develops more adaptive meta-beliefs, since GAD is known to respond only modestly to conventional CBT [31].

The bCBT delivery consisted of 15 weekly alternating face-to-face sessions (8) and online sessions with asynchronous therapeutic feedback (7). Online sessions were provided on a web-based treatment platform (Minddistrict, www.minddistrict.com), accessible through password-protected accounts. Online sessions contained text-based information and videos in which a therapist explained the theory, followed by exercises and homework assignments with examples from fictional patients. Feedback involved text-based messages from the therapist about the content of the online exercises performed by the patient and about treatment progress. ftfCBT entailed 15 weekly face-to-face sessions with similar content to the sessions of the bCBT protocol.

### Measures

Online questionnaires were administered at baseline, at week 7 (mid-treatment), at week 15 (post-treatment) and at one-year follow-up (see S4 Appendix for an overview of measures administered at each assessment interval). All questionnaires were self-administered, except for the diagnostic interview at baseline. The Dutch versions of the questionnaires were used. Our original study protocol specified that follow-up data would be collected after 67 weeks, one year after the post-treatment assessment, but for pragmatic reasons (funder requirements in terms of final deadline), the time frame was adjusted to 52 weeks. Furthermore, to reduce burden on participants, quality of life was measured only by the EuroQol (EQ-5D-5L) [32] and not by the Short Form Health Survey (SF-36) [33] as well as both measure quality of life and anxiety severity was measured only by the Beck Anxiety Inventory (BAI) and not by the disorder-specific questionnaires as the overall sample size would be too small for robust subgroup-analyses. That means the Short Form Health Survey (SF-36) and the disorder-specific questionnaires were not administered. These changes were made prior to trial commencement (see our published study protocol [29]).

Demographic characteristics such as age, gender, education and employment were collected at baseline. Diagnoses were assessed at baseline with the Structured Clinical Interview for DSM-IV Axis I Disorders (SCID-I) [26] or the Mini-International Neuropsychiatric Interview, Plus version (MINI-Plus) [27, 28].

#### Acceptability

We distinguished four aspects of acceptability: treatment preference, treatment adherence, therapeutic working alliance and treatment satisfaction. *Treatment preference* was assessed by asking participants to indicate their preference for bCBT or ftfCBT at baseline, prior to randomisation. *Treatment adherence* concerns the extent to which participants were exposed to the content of the interventions, as measured in three ways: (i) the percentage of completed prescribed sessions; (ii) the percentage of participants that finished treatment, defined as completing at least 15 sessions as described in the protocol or dropping out due to remission; and (iii) the duration of treatment in weeks.

The Revised Short Version of the Working Alliance Inventory (WAI-SR) [34, 35] was administered halfway through treatment to both patients and therapists to rate the quality of the *therapeutic alliance*. The WAI-SR has excellent psychometric properties [35]. To evaluate *treatment satisfaction* at post-treatment, we administered the Client Satisfaction Questionnaire-8 (CSQ-8) [36, 37] and, additionally for the participants randomised to bCBT, the System Usability Scale (SUS) [38, 39]. Both the CSQ-8 and the SUS scales have demonstrated reliability and validity [37, 39].

#### Effectiveness

Clinical outcome variables were assessed at baseline, at post-treatment (15 weeks) and at one-year follow-up 52 weeks after baseline. The *primary clinical outcome* was presence and severity of anxiety symptoms, as assessed with the Beck Anxiety Inventory (BAI) [40]. It contains 21 questions and total scores range between 0 and 63, with higher scores indicating more anxiety. The BAI is a reliable and well validated self-rated measure of anxiety symptoms [41].

*Secondary clinical outcome variables* included depressive symptoms, general psychopathology, mastery, social and work functioning, and quality of life, likewise assessed at baseline, post-treatment and follow-up. Presence and severity of depressive symptoms were assessed using the Beck Depression Inventory-II (BDI-II) [42, 43], which has highly acceptable psychometric properties [42]. Severity of general psychopathology was evaluated by the Brief Symptom Inventory (BSI) [44, 45], whose psychometric properties are good [45]. The five-item version of the Mastery Scale [46] was administered to assess perceived control of a person’s own life; it is a psychometrically valid instrument [46]. The Work and Social Adjustment Scale (WSAS), with adequate psychometric properties [47], is a measure of impaired functioning; it assesses the impact of a person’s mental health problems on their ability to function in terms of work, home management, social leisure, private leisure and personal or family relationships.

#### Quality of life

To estimate utilities the EuroQol (EQ-5D-5L) was administered [32]. We applied the Dutch tariff [48] to calculate the utilities. The EuroQol consists of five questions that gauge mobility, self-care, daily activities, pain and mood. It is the preference-based generic instrument for measuring health-related quality of life (HR-QoL) that is recommended by the Dutch guidelines for economic evaluations in healthcare and it has good psychometric properties [49]. Quality-adjusted life-years (QALYs) were calculated using the area-under-the-curve method (AUC) [50]. The health state descriptions were linked to empirical valuations of the Dutch general public, allowing utilities to be computed.

#### Costs

Costs were assessed at baseline, post-treatment and one-year follow-up using the Treatment Inventory Cost in Psychiatric Patients instrument (TiC-P) [51]. Costs can be determined from several perspectives. In this study we calculated costs for both the healthcare perspective (including direct medical costs) and the societal perspective (including direct medical costs, patient costs and productivity costs). Direct medical costs consist of costs for the use of healthcare services; patient costs consist of travel costs; productivity costs include costs arising from absenteeism and presenteeism.

In the TIC-P, a maximum recall period of 15 weeks was used and cumulative costs over the one-year study period were estimated using linear interpolation. In accordance with the TiC-P manual, a specific item on the service use accountable to the bCBT intervention was added to the default TiC-P. Direct medical costs, patient costs and productivity costs were valued using Dutch indexed standard reference prices of 2018 (see S5 Appendix) [52]. The friction cost method was applied to estimate productivity losses in paid work [53].

### Sample size and power

The trial was powered to investigate the joint distribution of costs and treatment effects [29]. We aimed to include 156 participants, with 78 in each condition, based on a power of 0.80 calculated by using the formula of Glick [54].

### Statistical analyses

Statistical analyses were conducted using the Statistical Package for the Social Sciences version 24.0 (IBM Corporation, Somers, NY, USA) and Excel (2013). The descriptive characteristics of the bCBT and ftfCBT groups and differences between study dropouts and study completers were compared using *t*-test for continuous variables and chi-square test for proportions.

#### Acceptability

Acceptability outcomes (treatment preference, treatment adherence, therapeutic working alliance, treatment satisfaction) were compared using *t*-test for continuous variables and chi-square test for proportions.

#### Effectiveness

Clinical outcomes were analysed on the basis of the intention-to-treat (ITT) principle. Linear mixed model (LMM) analyses with restricted maximum likelihood (REML) were conducted to evaluate differences in symptom reduction between the bCBT and ftfCBT groups at post-treatment and one-year follow-up. The linear mixed models were adjusted for baseline scores, because using analysis of covariance the estimate of the intervention effect is not affected by baseline differences and more statistical power to detect a treatment effect is achieved [55]. The LMM approach has the ability to handle missing data, as it uses all available data to estimate parameters for missing values and can account for the correlation between repeated measures [56]. A separate model was estimated for each of the outcome measures. A Bonferroni-Holm correction was applied to adjust for repeated comparisons, yielding a significance level of *p* = 0.01 (.05 / 5) [57].

Effect sizes (Cohen’s *d*) were calculated both within and between groups from estimated means and observed pooled standard deviations. The within-group effect size was computed as *d* = Mean_diff_ / SD_diff_, where Mean_diff_ is the mean difference between the values at pre-test and at post-test or follow-up and SD_diff_ = √(SD^2^_pre_ + SD^2^_post_ − 2*r*SD_pre_SD_post_), with *r* being the correlation between the pre-test and the post-test or follow-up values. The between-group effect size was computed as *d* = Mean_diff_ / SD_pooled_, where Mean_diff_ is the mean difference between bCBT scores and ftfCBT scores.

#### Cost-effectiveness

Yearly costs and QALYs were modelled using generalized linear models (GLM), that can manage skewness of data [58]. Missing utility values and costs at each time point were imputed using multiple imputation by predictive mean matching [59, 60]. For the estimation of costs, a log link and gamma family were used adjusting for age and baseline costs. For the estimation of QALYs a log link and gaussian family were used adjusting for age and baseline utility. The cost-effectiveness analysis was conducted by calculating the incremental costs per incremental QALY over the one-year follow-up period, resulting in the incremental cost-effectiveness ratio (ICER). The formula (C^1^-C^2^)/(E^1^-E^2^) was used, where (C^1^-C^2^) is the difference in costs between bCBT and ftf CBT, and (E^1^-E^2^) is the difference of the average effectiveness of bCBT and ftf CBT [61].

The ICER was estimated from a healthcare and societal perspective. The latter included the direct medical costs, patient costs and productivity costs, while the healthcare perspective is limited to the direct medical costs. Costs and effects are not discounted as the time-horizon of the current study did not exceeded 12 months follow-up.

Standard errors around the GLM coefficients were used to explore the uncertainty of the ICER. For this purpose, 10,000 populations were simulated using non-parametric bootstrapping. Cholesky decomposition [62] was used to retain the correlations between the parameters. The simulated results were plotted in a CE-plane [63], on which uncertainty around incremental costs and incremental effects was displayed graphically by the scatter of ICERs. From a cost-effectiveness perspective, the southeast quadrant indicates superior treatment effects and lower costs for bCBT in comparison with ftfCBT. If the ICER falls into this quadrant this indicates dominance of bCBT over ftfCBT and should lead to a positive reimbursement decision. The northwest indicates reduced treatment effects and higher costs for bCBT, thus leading to a negative reimbursement decision. ICERs in the two remaining quadrants indicate either that bCBT is less expensive but also less effective (southwest quadrant) or more effective but also more expensive (northeast quadrant). The cost-effectiveness of the latter depends on the threshold of the cost-effectiveness ratio. For the Netherlands the threshold is €20,000 to €80,000 depending on the severity of the disease. The uncertainty in the cost-effectiveness analysis was assessed using bootstrapping in Excel, with 10,000 iterations. This was expressed in a cost-effectiveness acceptability curve. The acceptability curve illustrates the probability that the cost-effectiveness ratio will be accepted for different cost limits [64].

## Results

### Study sample and attrition

**Fig 1:**
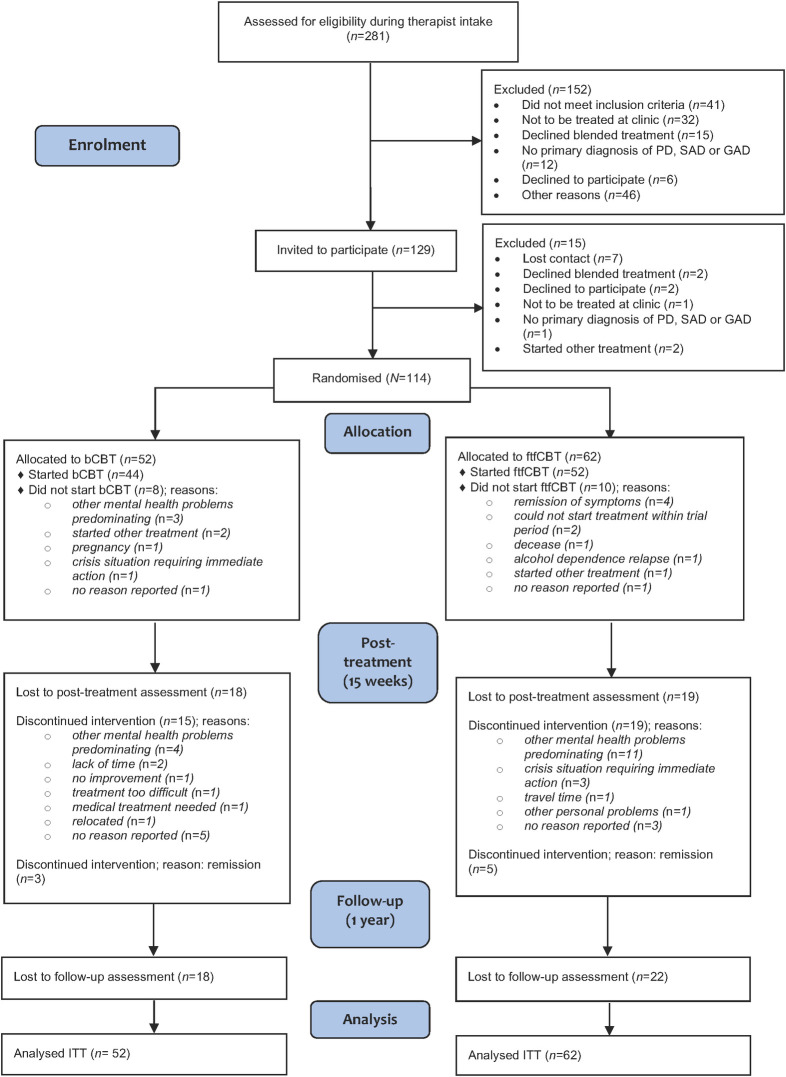
CONSORT flow diagram of participants.

A total of 281 participants were assessed by mental health professionals during the intake procedure; 129 eligible candidates were invited for a diagnostic interview and 114 were randomised to either bCBT (*n* = 52) or ftfCBT (*n* = 62; for details, see Fig 1). Demographic data of the included participants are presented in Table 1. The mean age of participants was 36.3 years (SD 10.6, range 19 to 69) and 60.5% were female (*n* = 69). Most patients had panic disorder as primary diagnosis (54.4%).

**Table 1:** Baseline characteristics of participants in bCBT and ftfCBT groups.

Characteristics	bCBT (*n* = 52)	ftfCBT (*n* = 62)	Total (*n* = 114)
Demographics			
*Age*, *mean (SD)*	36.0 (10.4)	36.5 (10.9)	36.3 (10.6)
*Female*, n *(%)*	26 (50.0)	43 (69.4)	69 (60.5)
*Higher education*,^*a*^ n *(%)*	16 (30.8)	14 (22.5)	30 (26.3)
*Employed*, n *(%)*	35 (67.3)	45 (72.6)	80 (70.2)
*Student*, n *(%)*	7 (13.5)	9 (14.5)	16 (14.0)
*Born in Netherlands*, n *(%)*	46 (88.5)	53 (85.5)	99 (86.8)
Taking psychotropic medication	29 (55.8)	38 (61.3)	67 (58.8)
Primary diagnosis, *n* (%)			
*Panic disorder*	27 (51.9)	35 (56.5)	62 (54.4)
*Social anxiety disorder*	12 (23.1)	13 (21.3)	25 (21.9)
*Generalised anxiety disorder*	13 (25.0)	14 (23.0)	27 (23.7)
Comorbidity, *n* (%)			
*Any comorbid disorder*	32 (61.5)	38 (61.3)	70 (61.4)
*Anxiety disorders*	16 (30.8)	15 (24.2)	31 (27.2)
*Mood disorders*	17 (32.7)	20 (32.3)	37 (32.5)
*Other disorders*	10 (19.2)	7 (11.3)	17 (14.9)

_bCBT: blended cognitive-behavioural therapy; ftfCBT: face-to-face cognitive-behavioural therapy; comorbid anxiety disorders: social phobia, panic disorder, agoraphobia, generalised anxiety disorder; comorbid mood disorders: major depressive disorder, dysthymia; other comorbid disorders: posttraumatic stress disorder, obsessive-compulsive disorder, eating disorder._^a^_Bachelor’s equivalent or higher._

The post-treatment assessments at 15 weeks were completed by 77 (67.5%) participants (bCBT *n* = 34, ftfCBT *n* = 43) and the one-year follow-up assessments by 74 (64.9%) participants (bCBT *n* = 34, ftfCBT *n* = 40). There was no significant difference in total study dropout between the two treatment groups, χ^2^ (1) = 0.78, *p* = 0.781. We tested for significant differences in demographic variables, primary diagnosis or presence of comorbidity between those who completed all post-baseline assessments and those who did not. Participants with missing data at one or more of those assessments (*n* = 51) were less likely to have a comorbid diagnosis, χ^2^ (2) = 4.84, *p* = 0.028.

### Acceptability

Queried prior to randomisation, patients expressed a slight preference for bCBT (54.4%) over ftfCBT (45.6%). The percentages with a bCBT preference (56.5% in the ftfCBT treatment group, 51.9% in the bCBT group) did not differ significantly between the groups, χ^2^ (1) = 0.23, *p* = 0.629.

Adherence in terms of the percentage of completed prescribed sessions was slightly but not significantly higher in the bCBT group, at 67.4% compared with 61.6% for the ftfCBT group (*t* = −0.515, *p* = 0.608). Thirty-one patients (59.6%) in the bCBT group and 32 patients (51.6%) in the ftfCBT group completed treatment (*t* = −0.795, *p* = 0.428). Treatment duration was shorter in the bCBT group, with an average of 14.4 weeks (range 0 to 56.4) compared with 16.1 weeks (range 0 to 67.7) for ftfCBT treatment (*t* = 0.796, *p* = 0.428).

The alliance assessment (WAI-SR) halfway through treatment was completed by 81 participants (71.1%) and 87 times (76.3%) by therapists. Participants in both groups reported high levels of working alliance on the WAI-SR, with a mean rating of 4.27 out of 5 (SD 0.69) in the bCBT group and 4.25 (SD 0.51) in the ftfCBT group. Therapists’ ratings were in a similar range, with scores of 4.32 (SD 0.37) in bCBT and 4.24 (SD 0.56) in ftfCBT. We found no significant difference between bCBT and ftfCBT in terms of WAI patient ratings (*t* = −0.111, *p* = 0.912) nor WAI therapist ratings (*t* = −0.304, *p* = 0.762), indicating no difference in working alliance between groups.

On average, participants in both groups reported high levels of treatment satisfaction. The mean scores on the CSQ-8 were 25.61 out of 32 (SD 4.21, range 8 to 32) for the bCBT group and 25.90 (SD 3.24) for the ftfCBT group, both lying between ‘somewhat satisfied’ (score 24) and ‘very satisfied’ (score 32). We found no significant difference between bCBT and ftfCBT in treatment satisfaction (*t* = 0.320, *p* = 0.750).

The online treatment platform was evaluated by patients randomised to the blended condition at an ‘above average’ score of 69.11 (SD 19.32) on the SUS.

### Effectiveness

Mean observed scores on primary and secondary clinical outcome measures at baseline, post-treatment and one-year follow-up are displayed in Table 2, accompanied by within-group and between-group effects. No statistically significant differences emerged between the bCBT group and the ftfCBT group in terms of decreased anxiety severity, either at post-treatment (*t* = −0.715, *p* = 0.477) or at follow-up (*t* = 1.702, *p* = 0.093). Within-group effect sizes from baseline to post-treatment were *d* = 0.73 for bCBT and *d* = 0.55 for ftfCBT and from baseline to follow-up *d* = 0.50 for bCBT and *d* = 1.00 for ftfCBT.

**Table 2:** Observed means and standard deviations for clinical outcome variables at baseline, post-treatment and one-year follow-up within each group, within-group effects and between-group effects based on estimated means.

		Blended CBT	Within-group effect size ^a^		Face-to-face CBT	Within-group effect size ^a^	Between-group comparison ^b^	Between-group effect size ^c^
Measure	*n*	Mean (SD)	Cohen’s *d* (95% CI)	*n*	Mean (SD)	Cohen’s *d* (95% CI)	*t* (*p*-value)	Cohen’s *d* (95% CI)
**Primary outcome**								
**Anxiety (BAI)**								
*Baseline*	51	27.90 (12.02)		62	27.15 (11.67)			
*Post-treatment*	34	17.18 (10.28)	0.73 (0.49, 0.97)	43	18.93 (11.55)	0.55 (0.34, 0.75)	−0.715 (0.477)	0.15 (−0.30, 0.60)
*1-year follow-up*	34	19.97 (13.12)	0.50 (0.25, 0.74)	40	14.28 (9.06)	1.00 (0.74, 1.26)	1.702 (0.093)	−0.38 (−0.84, 0.09)
**Secondary outcomes**								
**Depression (BDI-II)**								
*Baseline*	52	23.98 (12.17)		62	24.00 (10.26)			
*Post-treatment*	34	16.50 (11.63)	0.53 (0.30, 0.76)	42	18.69 (10.76)	0.42 (0.21, 0.62)	−0.801 (0.425)	0.16 (−0.30, 0.61)
*1-year follow-up*	32	15.69 (11.13)	0.72 (0.45, 0.98)	39	14.69 (9.44)	0.59 (0.36, 0.82)	0.203 (0.840)	−0.04 (−0.51, 0.43)
**General psychopathology (BSI)**								
*Baseline*	52	1.43 (0.72)		62	1.36 (0.67)			
*Post-treatment*	34	0.97 (0.63)	0.67 (0.44, 0.91)	42	0.95 (0.66)	0.50 (0.29, 0.70)	−0.130 (0.897)	0.02 (−0.43, 0.48)
*1-year follow-up*	32	1.00 (0.66)	0.63 (0.37, 0.89)	39	0.75 (0.61)	0.98 (0.72, 1.24)	1.339 (0.185)	−0.27 (−0.74, 0.20)
**Mastery (Mastery Scale)**								
*Baseline*	52	14.90 (4.45)		62	14.26 (4.39)			
*Post-treatment*	34	16.12 (4.20)	−0.26 (−0.48, −0.05)	42	16.05 (4.70)	−0.42 (−0.6, −0.22)	−0.290 (0.773)	0.05 (−0.40, 0.51)
*1-year follow-up*	32	15.56 (4.96)	−0.13 (−0.37, 0.10)	39	17.38 (4.78)	−0.63 (−0.86, −0.40)	−2.329 (0.023)	0.48 (0.01, 0.96)
**Work and social adjustment (WSAS)**								
*Baseline*	52	23.00 (10.22)		62	23.90 (9.11)			
*Post-treatment*	34	17.47 (9.61)	0.65 (0.41, 0.88)	42	18.95 (10.02)	0.35 (0.16, 0.55)	−0.751 (0.455)	0.15 (−0.31, 0.60)
*1-year follow-up*	32	16.34 (11.21)	0.66 (0.40, 0.92)	39	17.97 (10.77)	0.48 (0.26, 0.71)	−0.775 (0.441)	0.17 (−0.03, 0.64)
**Quality of life (EQ-5D utility scores)**								
*Baseline*	52	0.55 (0.28)		62	0.53 (0.26)			
*Post-treatment*	34	0.69 (0.20)	−0.47 (−0.69, −0.25)	43	0.61 (0.25)	−0.28 (−0.47, −0.08)	1.235 (0.220)	−0.24 (−0.69, 0.21)
*1-year follow-up*	34	0.69 (0.27)	−0.40 (−0.64, −0.16)	40	0.71 (0.25)	−0.60 (−0.83, −0.37)	−0.498 (0.620)	0.11 (−0.35, 0.56)

* _Note, Bonferroni-Holm corrected significance level is *p* = 0.01._

_Abbreviations: bCBT: blended cognitive-behavioural therapy; ftfCBT: face-to-face cognitive-behavioural therapy; BAI: Beck Anxiety Inventory; BDI-II: Beck Depression Inventory-II; BSI: Brief Symptom Inventory; WSAS: Work and Social Adjustment Scale; EQ-5D-5L: EuroQol._

^a^_Within-group effect sizes were calculated based on estimated means from the linear mixed model using raw differences._

^b^_Between-group comparisons were based on estimated means from the linear mixed model with baseline adjustment._

^c^_Between-group effect sizes were calculated based on estimated means from the linear mixed model with baseline adjustment._

Separate linear mixed model analyses revealed no significant effects of treatment condition at post-treatment or follow-up on secondary outcomes: depressive symptoms, general psychopathology, mastery, work and social functioning and quality of life. Within-group effect sizes at post-treatment and follow-up ranged from *d* = 0.13 to *d* = 0.98.

### Cost-effectiveness

The results from the cost-effectiveness analyses are presented in Table 3. Multiple imputation (cost data: 35.1% imputed, QALY data: 32,7% imputed) followed by modelled simulations yielded average direct medical costs of €3758 for bCBT and €3841 for ftfCBT over the one-year study period. Direct medical costs were statistically significantly lower in the bCBT group (mean -83,78, 95% CI -96,96 to -70,61, p<0.001). Societal costs were €10945 for bCBT and €10937 for ftfCBT. Differences were not statistically significant (mean 26,46, 95% CI -26,46 to 42,71, p>0.1). Total costs based on available data over the treatment period and the one-year follow-up period are included in S6 Appendix. The average QALYs over the one-year study period were 0.66 for bCBT and 0.62 for ftfCBT. QALYs were statistically significantly higher in the bCBT group (mean 0.037, 95% CI 0.036 to 0.038, p<0.001). This resulted in a dominant ICER from the healthcare perspective (€-2257 per QALY) and an ICER of €219 per QALY from the societal perspective.

**Table 3:** Results of cost-effectiveness analyses.

	Incremental costs, Eur, (95% CI)	Incremental effects, quality-adjusted life year (95% CI)	ICER, mean	Distribution over the ICER plane (%)			
				NE	NW	SE	SW
**Healthcare perspective**	€-83,78 (-96,96 to -70,61)	0.037 (0.036 to 0.038)	Dominant (€-2257)	37.6%	6.8%	46.6%	9.0%
**Societal perspective**	€8,13 (-26,46 to 42,71)	0.037 (0.036 to 0.038)	€219	41.8%	7.7%	42.3%	8.2%

* _Note, ICER: incremental cost-effectiveness ratio. Plane distribution: NE: more expensive, more effective; NW: more expensive, less effective; SE: less expensive, more effective; SW: less expensive, less effective._

**Fig 2:**
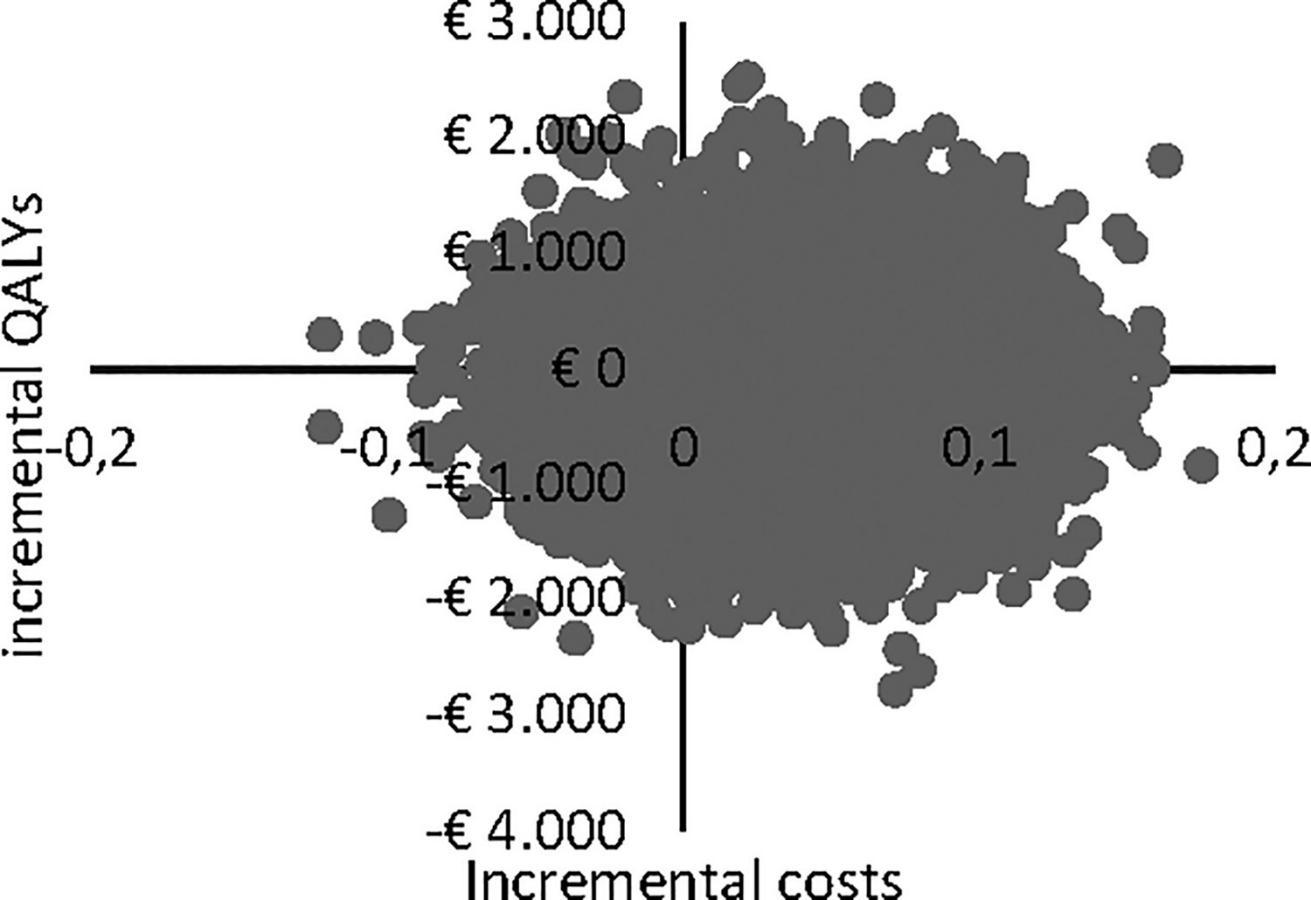
CE plane for healthcare perspective.

**Fig 3:**
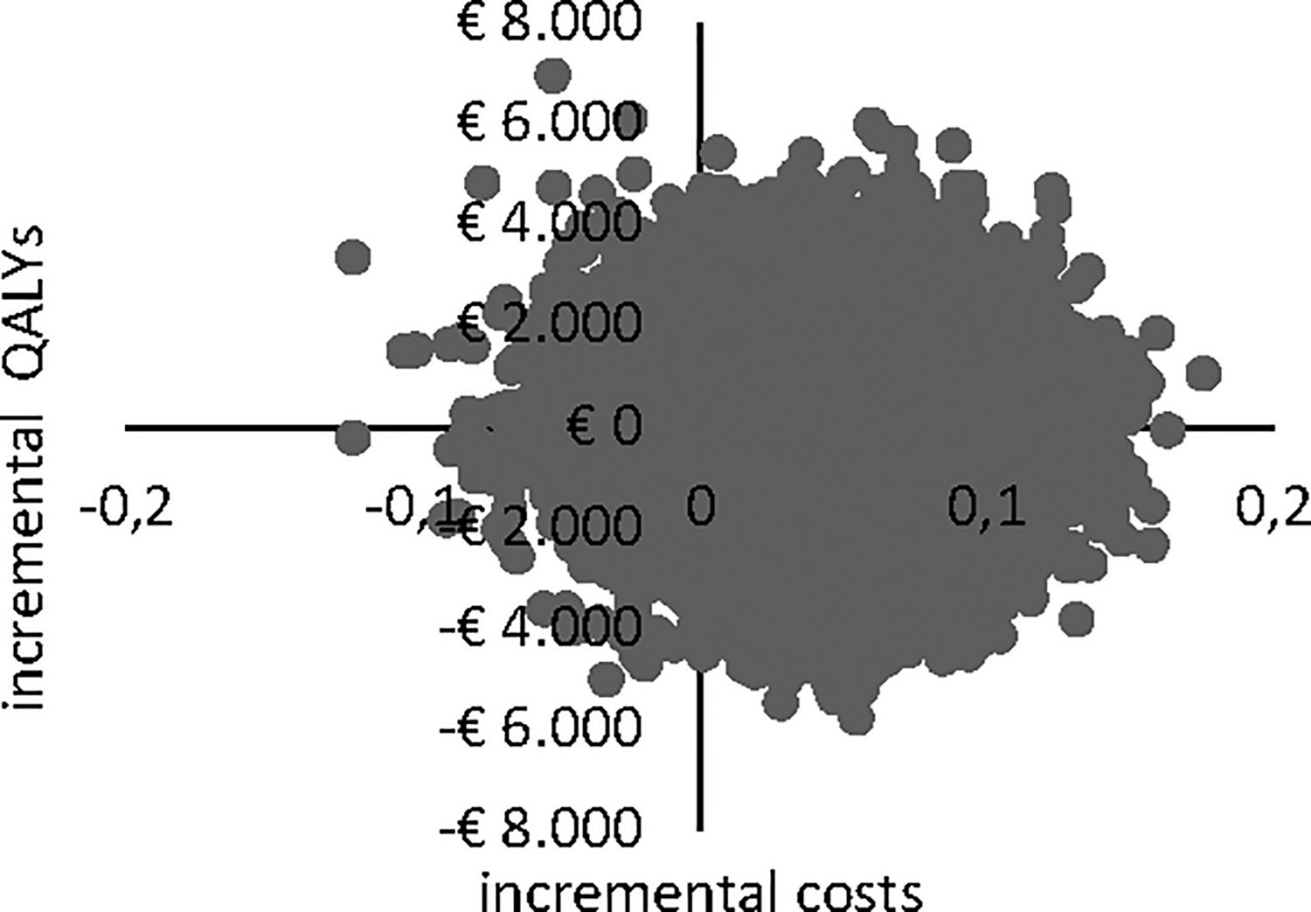
CE plane for societal perspective.

Uncertainty in cost and effect estimates is shown in cost-effectiveness planes (CE planes, Figs 2 and 3). The CE planes show that the greatest numbers of ICERs were situated in the southeast quadrant of the CE plane, both in the healthcare perspective (46.6%) and in the societal perspective (42.3%), indicating lower costs for bCBT as well as a superior effect in terms of quality of life. Another 37.6% were in the northeast quadrant from the healthcare perspective and 41.6% from the societal perspective respectively. From the health care perspective 6.8% and 9.0% of the estimates were in respectively in the north- and southwest quadrant. From the societal perspective these figures were 7.7% and 8.2%.

**Fig 4:**
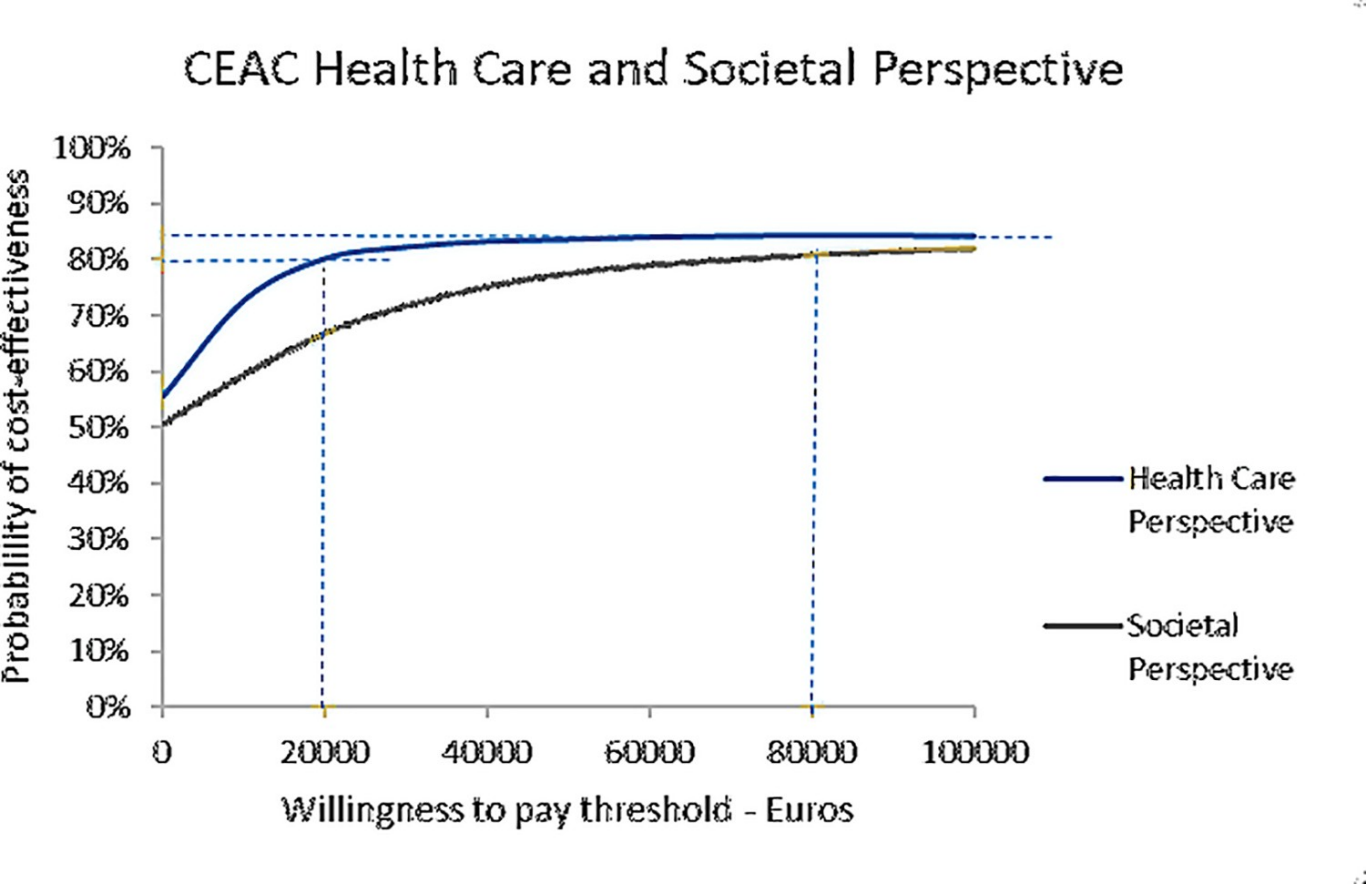
Cost acceptability curves from the societal perspective and health care perspective.

Determining the acceptability of the treatments, we calculated the proportion of ICERS that were below the threshold of 20,000 and 80,000 per QALY. The threshold is the willingness of society to pay and was varied as this is the common range for the Netherlands. The thresholds and the proportion of ICERS were subsequently plotted in the cost acceptability curve, see Fig 4. The figure shows that from a health care perspective, at a threshold of 20,000 Euro/QALY, the probability that the ratio is acceptable is more than 80%. Taking a societal perspective, the percentage that the intervention is acceptable was 67%. At a threshold of 80,000 Euro/QALY the intervention was acceptable more than 80% from both perspectives.

## Discussion

Blended treatment for anxiety disorders, which integrates face-to-face therapy and Internet-based therapy, has not yet been rigorously studied. To our knowledge, RCTs investigating effectiveness and costs are lacking. This study is the first to assess the acceptability, effectiveness and cost-effectiveness of bCBT vis-à-vis ftfCBT in outpatients receiving specialised mental health care who have been diagnosed with panic disorder, social anxiety disorder or generalised anxiety disorder.

Our findings on acceptability indicate that bCBT is an acceptable treatment option for patients in specialised mental health care in terms of treatment preference, adherence, therapeutic alliance and treatment satisfaction. Over half (54.9%) of the participants would have preferred to start with bCBT above ftfCBT. Although that is the treatment preference of patients who consented to take part in the current study, and hence not a fully representative finding for all patients in specialised mental health care, it does reveal that a considerable desire for the blended treatment format exists in that population. Therapeutic alliance and treatment satisfaction were high for both bCBT and ftfCBT patients, and treatment adherence rates were comparable for both groups.

With regard to effectiveness in reducing anxiety symptoms, we found no significant differences between bCBT and ftfCBT at post-treatment (*t* = −0.715, *p* = 0.477) nor at one-year follow-up (*t* = 1.702, *p* = 0.093). Both groups exhibited moderate to large within-group effect sizes (range of *d*: 0.50 to 1.00). Moreover, no significant differences between the groups were found in terms of effects on depressive symptoms, general psychopathology, sense of control (mastery), work and social functioning or quality of life, with within-group effect sizes ranging from small to large (range of *d*: 0.13 to 0.98).

In the current study online sessions partially replaced face-to-face sessions in the blended treatment. Other studies have investigated iCBT applied as an adjunctive to ftfCBT.

Our clinical findings appear to be in line with results from those studies. For example, Nordgreen and colleagues conducted an RCT (*N* = 173) whereby iCBT and ftfCBT for panic disorder and social anxiety disorder were combined in a stepped-care format, with a face-to-face psychoeducation session as first step, online treatment (9 or 10 sessions) as second step and face-to-face treatment (12 sessions) as final step [65]. The stepped-care variant was compared with ftfCBT (12 sessions). No significant differences in the reductions of anxiety symptoms and depressive symptoms were found between the groups at post-treatment and one-year follow-up, and within-group effect sizes were moderate to large. Comparability with our study is limited, however, as the stepped-care format consisted of iCBT as an add-on prior to ftfCBT. Moreover, treatment attrition in the stepped-care group was high (41.2%), with the majority dropping out before starting the face-to-face treatment, meaning that they only received online iCBT. In a pilot study (*N* = 36) by Bruinsma and colleagues [23], a combination of 9 ftfCBT sessions supplemented with 3 iCBT sessions was compared with 12-session ftfCBT. They found no significant between-group differences at post-treatment in terms of improvement rates on panic-related symptoms and general functioning, with moderate to large within-group effect sizes.

Our cost analysis showed that societal costs were relatively larger than direct medical costs in both groups. This may be due to relatively low treatment costs and a large proportion of patients of working age. This finding is in line with literature that showed that productivity costs are commonly responsible for the majority of the total costs [3, 66]. These results highlight the substantial societal burden of anxiety disorders and the importance of making CBT for anxiety disorders more accessible. Further findings have shown that the costs for providing treatment would be compensated within two to five years by increased productivity resulting from the intervention [67].

The acceptability curves in the current study revealed that bCBT was expected to be a cost-effective intervention. While bCBT point estimates suggest slightly lower healthcare and slightly higher societal costs than ftfCBT over the one-year study period, the probabilistic results suggest a high probability of cost-effectiveness taking a threshold of €20.000 from both perspectives. In contrast, in a naturalistic study by Kenter and colleagues treatment time and costs increased for bCBT relative to ftfCBT [68]. In this study, no treatment protocol or clear guidelines on how to apply blended treatment were available and therapists turned out to have provided online sessions on top of face-to-face sessions resulting in longer treatment durations. This marked contrast to our trial possibly explains the difference in outcomes.

A strength of this study is that it is the first randomised controlled trial to explore acceptability, effectiveness and cost-effectiveness by comparing equal-intensity bCBT and ftfCBT for anxiety disorders in routine outpatient specialised mental health care. In addition, participants in our study appear to be a clinically representative sample, in view of the large proportion of patients with comorbid disorders, lower education levels and severe anxiety symptoms at baseline, in comparison with self-referred samples recruited from the community [12]. Clinical representativeness is also reflected by the high productivity costs and the low scores on measurements of work, social functioning and quality of life; disability and decreased productivity are common among patients with severe anxiety disorders [69, 70]. Although patients in both groups exhibited improvement on these scores at post-treatment and follow-up, the scores remained relatively low in comparison with those in the general healthy population [47, 71]. That further demonstrates the severity and complexity of problems in the current study sample.

Some limitations are associated with the present study. First, because the sample size was smaller than expected, only initial indications of the cost-effectiveness of bCBT in comparison with ftfCBT could be explored. Due to financial and time limits, only 114 participants rather than the intended 156 were included. However, it might be noted that such a sample size is considerable for routine specialised mental health care populations. Sample size and power challenges are common issues in trials investigating both clinical and cost-effectiveness [72]. In line with recommendations for dealing with such issues [73], the uncertainty was presented in cost-effectiveness planes. Another limitation lies in the substantial study dropout rate (35.1% at one-year follow-up), which was not considered in the power calculation. This dropout seems to reflect the reality of trials conducted in routine mental health care, as comparable rates were found in earlier clinical trials comparing iCBT with ftfCBT [65, 74–77]; it could not be prevented by our e-mail and telephone reminders. To handle missing data, a linear mixed model was used to analyse clinical effectiveness, and imputations were used for the cost-effectiveness analyses.

In addition, participants and therapists could not be blinded to treatment allocation. That was inevitable given the nature of this trial, but it may have affected results. For example, participants who know they are in the ‘experimental condition’ are more likely to provide biased effectiveness assessments than blinded participants; blinded therapists are less likely than unblinded therapists to provide additional treatment interventions [78].

Finally, we used the EQ-5D for measuring quality of life. In recent years, the usefulness of the EQ-5D to measure mental health related quality of life has been questioned [79, 80]. Other questionnaires are available that include more dimensions of quality of life relevant to populations of people with mental health problems. For example, the more recently developed Assessment of Quality of Life–Eight Dimension Scale (AQoL-8D) [81] might serve as an alternative for the EQ-5D. However, validity of this instrument has not been tested in the Dutch population, which is one of the reasons that the EQ-5D is the recommended questionnaire for economic evaluations in the Dutch context [82]. Furthermore, the EQ-5D is reasonably responsive in patients with anxiety disorders [83] and thus seems suitable in the current study. Nevertheless, other available instruments to evaluate mental health related quality of life should be considered in future research, especially when research is focusing on mental disorders such as schizophrenia and bipolar disorder [84], and validation of the AQoL-8D for the Dutch population would be desirable.

In sum, our results suggest that bCBT is an acceptable approach for patients with anxiety disorders in specialised mental health care settings. We found no indications that its clinical effectiveness differs from that of ftfCBT. Moreover, bCBT is expected to be a cost-effective alternative to ftfCBT.

## Supporting information

S1 Appendix CONSORT checklist. (DOC)

S2 Appendix CHEERS checklist. (PDF)

S3 Appendix Study protocol. (DOCX)

S4 Appendix Overview of measures. (DOCX)

S5 Appendix Unit costs. (DOCX)

S6 Appendix Total costs based on observed means. (DOCX)
