# Supplementary material for: Acceptability, effectiveness and cost-effectiveness of blended cognitive-behavioural therapy (bCBT) versus face-to-face CBT (ftfCBT) for anxiety disorders in specialised mental health care: A 15-week randomised controlled trial with 1-year follow-up
Source: PLoS One. 2021 Nov 12;16(11):e0259493. doi: 10.1371/journal.pone.0259493 (PMC8589191; doi:10.1371/journal.pone.0259493)
Supplement: S3 Appendix — (DOCX) [file pone.0259493.s003.docx]

**Research protocol**

**Cost-utility and cost-effectiveness of blended eHealth treatment for severe anxiety disorders in secondary mental health care.**

**Version 5, 22-9-2015**

**PROTOCOL TITLE**

Cost-utility and cost-effectiveness of blended eHealth treatment for severe anxiety disorders in secondary mental health care’

| **Protocol ID** | ***CWO-2014-12*** |
| --- | --- |
| **Short title** | ***Health care efficiency for blended eHealth treatment for anxiety*** |
| **EudraCT number** | ***Not Applicable (NA)*** |
| **Version** | ***4*** |
| **Date** | ***3-8-2015*** |
| Coordinating investigator/project leader | ***Prof. Dr. Heleen Riper VU University Amsterdam, Department of Clinical Psychology***  ***Van der Boechorststraat 1, 1081 BT, Amsterdam 020-5982619***  ***Dr .J.P.F. Koning***  ***Psychiatrist and senior investigator GGZ Altrecht, Psychiatry Department Mimosastraat 2-4,3551 DC Utrecht***  ***030-3201001*** |
| Principal investigator(s) (in Dutch: hoofdonderzoeker/ uitvoerder)  *<Multicenter research: per site>* | ***Dr. J.P.F. Koning  Pro Persona geestelijke gezondheidszorg Siependaallaan 3, 4003 LE Tiel***  ***0344 656 111***  ***Prof. dr. A.J.L.M. van Balkom***  ***GGZinGeest VUmc en GGZ inGeest***  ***A.J.Ernststraat 1187***  ***1081 HL Amsterdam***  ***020-78845491070***  ***Linda Willems***  ***GGZ Oost Brabant***  ***Joannes Zwijsenlaan 123***  ***5342BT Oss***  ***0413 - 84 6800***  ***Dr. Geert Uijterwaal***  ***Joannes Zwijsenlaan 123***  ***5342 BT Oss***  ***0412 – 847000 / 06 – 53143888*** |
|  |  |
| **Sponsor (in Dutch: verrichter/opdrachtgever)** | **VU University Amsterdam** |
|  |  |
| **Subsidising party** |  |
| **Independent expert (s)** | ***Dr. Afke Terwisscha***  ***GGZ Altrecht***  ***Psychiatrist Mimosastraat 2-4,3551 DC Utrecht***  ***030-3103100***  ***Dr. Anneke van Schaik GGZ inGeest***  ***Psychiatrist AJ Ernststraat 1187 1081 HL Amsterdam 020-7884534*** |
|  |  |
|  |  |
| **Laboratory sites <*if applicable*>** | ***Not applicable (NA)*** |
|  |  |
| **Pharmacy <*if applicable*>** | ***Not applicable (NA)*** |
|  |  |

**PROTOCOL SIGNATURE SHEET**

| **Name** | **Signature** | **Date** |
| --- | --- | --- |
| **Sponsor or legal representative:**  ***<please include name and function>***  ***<For non-commercial research,>***  **Head of Department:**  ***<include name and function>*** | ***Prof. Dr. Pim Cuijpers (Head of the Department of Clinical Psychology, VU University Amsterdam)*** |  |
| **[Coordinating Investigator/Project leader/Principal Investigator]:**  ***<please include name and function>*** | ***Prof. Dr. Heleen Riper VU University Amsterdam, Department of Clinical Psychology***  ***Van der Boechorststraat 1, 1081 BT, Amsterdam 020-5982619***  ***Dr .J.P.F. Koning***  ***Psychiatrist and senior investigator GGZ Altrecht, Psychiatry Department Mimosastraat 2-4,3551 DC Utrecht***  ***030-3201001*** |  |

**TABLE OF CONTENTS**

1. INTRODUCTION AND RATIONALE 8

2. OBJECTIVES 9

3. STUDY DESIGN 10

4. STUDY POPULATION 11

4.1 Population (base) 11

4.2 Inclusion criteria 11

4.3 Exclusion criteria 11

4.4 Sample size calculation 11

5. TREATMENT OF SUBJECTS 12

5.1 Investigational product/treatment 12

5.2 Use of co-intervention (if applicable) 12

5.3 Escape medication (if applicable) 12

6. INVESTIGATIONAL PRODUCT 13

6.1 Name and description of investigational product(s) 13

6.2 Summary of findings from non-clinical studies 13

6.3 Summary of findings from clinical studies 13

6.4 Summary of known and potential risks and benefits 13

6.5 Description and justification of route of administration and dosage 13

6.6 Dosages, dosage modifications and method of administration 13

6.7 Preparation and labelling of Investigational Medicinal Product 13

6.8 Drug accountability 13

7. NON-INVESTIGATIONAL PRODUCT 14

7.1 Name and description of non-investigational product(s) 14

7.2 Summary of findings from non-clinical studies 14

7.3 Summary of findings from clinical studies 14

7.4 Summary of known and potential risks and benefits 14

7.5 Description and justification of route of administration and dosage 14

7.6 Dosages, dosage modifications and method of administration 14

7.7 Preparation and labelling of Non Investigational Medicinal Product 14

7.8 Drug accountability 14

8. METHODS 15

8.1 Study parameters/endpoints 15

8.1.1 Main study parameter/endpoint 15

8.1.2 Secondary study parameters/endpoints (if applicable) 15

8.1.3 Other study parameters (if applicable) 15

8.2 Randomisation, blinding and treatment allocation 15

8.3 Study procedures 15

8.4 Withdrawal of individual subjects 15

8.4.1 Specific criteria for withdrawal (if applicable) 15

8.5 Replacement of individual subjects after withdrawal 15

8.6 Follow-up of subjects withdrawn from treatment 15

8.7 Premature termination of the study 15

9. SAFETY REPORTING 16

9.1 Section 10 WMO event 16

9.2 AEs, SAEs and SUSARs 16

9.2.1 Adverse events (AEs) 16

9.2.2 Serious adverse events (SAEs) 16

9.2.3 Suspected unexpected serious adverse reactions (SUSARs) 17

9.3 Annual safety report 18

9.4 Follow-up of adverse events 19

9.5 [Data Safety Monitoring Board (DSMB) / Safety Committee] 19

10. STATISTICAL ANALYSIS 20

10.1 Primary study parameter(s) 20

10.2 Secondary study parameter(s) 20

10.3 Other study parameters 20

10.4 Interim analysis (if applicable) 20

11. ETHICAL CONSIDERATIONS 21

11.1 Regulation statement 21

11.2 Recruitment and consent 21

11.3 Objection by minors or incapacitated subjects (if applicable) 21

11.4 Benefits and risks assessment, group relatedness 21

11.5 Compensation for injury 21

11.6 Incentives (if applicable) 22

12. ADMINISTRATIVE ASPECTS, MONITORING AND PUBLICATION 23

12.1 Handling and storage of data and documents 23

12.2 Monitoring and Quality Assurance 23

12.3 Amendments 23

12.4 Annual progress report 24

12.5 End of study report 24

12.6 Public disclosure and publication policy 25

13. STRUCTURED RISK ANALYSIS 26

13.1 Potential issues of concern 26

13.2 Synthesis 27

14. REFERENCES 27

**LIST OF ABBREVIATIONS AND RELEVANT DEFINITIONS**

| **ABR** | **ABR form, General Assessment and Registration form, is the application form that is required for submission to the accredited Ethics Committee (In Dutch, ABR = Algemene Beoordeling en Registratie)** |
| --- | --- |
| **AE** | **Adverse Event** |
| **AR** | **Adverse Reaction** |
| **CA** | **Competent Authority** |
| **CCMO** | **Central Committee on Research Involving Human Subjects; in Dutch: Centrale Commissie Mensgebonden Onderzoek** |
| **CV** | **Curriculum Vitae** |
| **DSMB** | **Data Safety Monitoring Board** |
| **EU** | **European Union** |
| **EudraCT** | **European drug regulatory affairs Clinical Trials** |
| **GCP** | **Good Clinical Practice** |
| **IB** | **Investigator’s Brochure** |
| **IC** | **Informed Consent** |
| **IMP** | **Investigational Medicinal Product** |
| **IMPD** | **Investigational Medicinal Product Dossier** |
| **METC** | **Medical research ethics committee (MREC); in Dutch: medisch ethische toetsing commissie (METC)** |
| **(S)AE** | **(Serious) Adverse Event** |
| **SPC** | **Summary of Product Characteristics (in Dutch: officiële productinfomatie IB1-tekst)** |
| **Sponsor** | **The sponsor is the party that commissions the organisation or performance of the research, for example a pharmaceutical**  **company, academic hospital, scientific organisation or investigator. A party that provides funding for a study but does not commission it is not regarded as the sponsor, but referred to as a subsidising party.** |
| **SUSAR** | **Suspected Unexpected Serious Adverse Reaction** |
| **Wbp** | **Personal Data Protection Act (in Dutch: Wet Bescherming Persoonsgevens)** |
| **WMO** | **Medical Research Involving Human Subjects Act (in Dutch: Wet Medisch-wetenschappelijk Onderzoek met Mensen** |

**SUMMARY**

**Rationale:** Anxiety disorders are highly prevalent disorders. They cause considerable human suffering and substantial societal and economic costs, estimated to be more than 1 billion euros annually. A possible way to make treatment more cost-effective lies in *blended care*. This type of treatment combines elements of both online and face-to-face cognitive behavioral therapy (CBT).

**Objective**: In this RCT, we will examine the health care efficiency of blended care treatment for patients with a severe anxiety disorder in outpatient specialized (secondary) mental health care. The results of this cost-effectiveness study will provide 1) an insight into the health-economical outcomes of ‘blended treatment’ 2) an indication whether blended treatment will add value when it is implemented in routine specialized mental healthcare settings, and 3) a good indication whether blended treatment for severe anxiety disorders in routine practice is advisable and feasible from the perspective of different stakeholders including patients, mental health service providers and health insurers.

**Study design:** A randomized controlled trial with two parallel conditions in which blended CBT will be compared to face tot face CBT treatment as usual (CBT-TAU), for anxiety disorders

**Study population:** Patients (≥18 years old) with a diagnosis of a severe anxiety disorder (panic disorder with or without agoraphobia, social phobia, and generalized anxiety disorder) who were referred to outpatient specialized mental health care services.

**Intervention**: The blended treatment consists of 12 to 20 CBT sessions, alternating between 6 to 10 face-to-face and 6 to 10 Internet sessions. Treatment as usual will consist of 12 to 20 face-to-face CBT sessions on average (CBT-TAU). (The number of sessions depends on the type of anxiety treatment).

**Main study parameters/endpoints:** The health-economic analyses combine clinical outcomes with cost estimates. Primary clinical outcomes are 1) recovery from*,* 2) changes in anxiety symptom severity, 3) quality-adjusted life years (QALY’s), and 4) health-related quality of life. Cost estimates are 1) costs of offering the treatments and 2) patients’ out-of-pocket costs (non-medical), such as the costs of travelling to the health services and the patients’ time costs of travelling, waiting and receiving treatment. Furthermore cost estimates include 3) costs stemming from health care uptake, including costs of medication and 4) costs stemming from productivity losses due to absenteeism and reduced efficiency while at work (presenteeism).

**Nature and extent of the burden and risks associated with participation, benefit and group relatedness:** Blended treatment is not considered to add risks to the treatment, compared to treatment as usual. The questionnaires can be considered a slight burden for patients in both conditions.

# INTRODUCTION AND RATIONALE

Anxiety disorders (1 year prevalence 10,1%, incidence 3,1%) are severe psychiatric disorders associated with a poor quality of life and substantial economic ramifications (Graaf R de, 2010; RIVM, 2013; Smit et al., 2006). In the Netherlands, the annual healthcare costs associated with anxiety disorders are estimated at €286 million, 45% (€128 million) of which is spent on secondary mental health care, to treat patients with more severe anxiety disorders. Costs due to absenteeism in anxiety disorders are estimated at €998 million per year (RIVM, 2013), exceeding those of depression which are estimated at €467.4 million (GGZNederland, 2010; RIVM, 2013; Smit et al., 2006) In 2007, the total disease burden caused by anxiety disorders was 202,000 DALY’s in the Netherlands, being third in the top ten list of medical disorders and having a higher cost impact than depression, diabetes or lung cancer (RIVM, 2013).

For the above reasons it is of paramount importance to deliver appropriate and efficient treatment in severe anxiety disorders to decrease the public health impact of these disorders. Anxiety disorders can be treated effectively with cognitive-behavioural therapies (CBT), whether or not combined with pharmacotherapy. CBT is regarded as treatment as usual (TAU) and recommended in national and international treatment guidelines (NICE; www.ggzrichtlijnen.nl). It has been shown that anxiety disorder patients in the Netherlands have a preference for non-pharmacological treatment. In addition, compared with pharmacotherapy, patients who achieve full remission after treatment with CBT are less likely to relapse when treatment has stopped (Bruce et al., 2005). However, less than half of the patients with anxiety disorders receives appropriate treatment (Bijl et al., 2003), due to anxiety related avoidance behaviour, stigmatisation, costs, and a perceived lack of availability of appropriate treatments (Reger and Gahm, 2009).

Internet interventions, especially internet-based cognitive behavioral treatment (iCBT), are seen as an important strategy for lowering the costs of the treatment of common mental health disorders (e.g. unipolar depression and anxiety disorders).

Internet based cognitive behavioural therapy programmes (iCBT) have been developed for most common anxiety disorders like panic disorder with or without agoraphobia, social phobia and generalized anxiety disorder, which have proven to be at least equally effective as face-to-face psychotherapy. Another advantage of iCBT is that it requires less therapist time and as a consequence is expected to be less costly than conventional CBT.

Studies show that anxiety treatment delivered via Internet is more effective than non-intervening and that it can be as effective as face-to-face treatment (Andersson et al., 2014; Andrews et al., 2010; Cuijpers et al., 2009; Haug et al., 2012; Lewis et al., 2012; Mayo-Wilson and Montgomery, 2013; Reger and Gahm, 2009; Spek et al., 2007). Importantly, studies investigating the cost-effectiveness of Internet-based depression and anxiety treatments suggest that these may also be more cost-effective than face-to-face treatment, but the number of studies is still scarce (e.g., (Gerhards et al., 2010; Hedman et al., 2014; Hedman et al., 2012; Lokkerbol et al., 2014; Nordgren et al., 2014; Smit et al., 2011; Tyrer et al., 2014; Warmerdam et al., 2010). Additionally, it should be noted that most study results are obtained among self-referred depressed individuals from the general population who participate in standalone Internet treatments (Riper, 2013).

Clinical and economical evaluations of treatment of anxiety via Internet among patients in routine primary care and specialized mental health care services are still scarce. A rather new treatment approach combines face-to-face treatment with Internet sessions into one integrated treatment. This is a so-called ‘blended’ treatment approach (Riper, 2013). Viewed from a cost-effectiveness perspective, blended treatment could possibly diminish the number of face-to-face contacts, increase self-management competencies of patients and thereby decrease the overall (direct) costs of depression treatment. This approach could also have a positive effect on waitlist periods, as therapists can take on more patients, reducing the number of patients that are waitlisted. Dutch mental health care organizations are ready to implement blended treatment, and they are motivated to do so by Dutch health policy makers and insurers (Bakker, 2013), because it is assumed that blended cognitive behavioural therapy (bCBT) and usual face-to-face CBT (CBT-TAU) are at least similarly clinically effective, and that bCBT can be offered at lesser costs. At present, however, little is known about the clinical and health-economic benefits of blended treatment.

In the current cost-effectiveness study, we will examine health care efficiency in a randomized controlled trial of bCBT vs. face-to-face CBT (CBT-TAU), among patients with a diagnosis of with a diagnosis of a severe anxiety disorder like panic disorder with or without agoraphobia, as social phobia or a generalized anxiety disorder. The proposed study is one of a series of projects in which we explore the potential of a ‘blended cognitive behavioral treatment‘ (bCBT) for anxiety and depression.

The study is funded through the healthcare efficiency funding program (Dutch: doelmatigheidsonderzoek) of the Netherlands Organization for Health Research and Development (ZonMw). In subprogram 2 of this funding program, ZonMw enables the investigation of health care efficiency of applied interventions.

# The results of this cost-effectiveness study will provide a) insight into the health-economical outcomes of ‘blended treatment’, b) indication whether blended treatment will add value when it is implemented in clinical settings, and c) insight in whether blended treatment for anxiety is advisable and feasible from the perspective of various stakeholders.2. OBJECTIVES

This cost-effectiveness study will compare blended cognitive behavioral treatment (bCBT) with standard face-to-face CBT (CBT-TAU) among patients with a diagnosis of an anxiety disorder referred to an outpatient specialized mental health care.

The main goal of the study is to explore the health-economic outcomes of bCBT in comparison to CBT-TAU, through cost-effectiveness analysis (CEA), cost-utility analysis (CUA) and a budget impact analysis (BIA), both from a societal and a healthcare perspective.

We assume that bCBT and CBT-TAU are similarly clinically effective, but that bCBT can be offered at lesser costs. Hence, we expect health-economic outcomes of bCBT to be favorable in comparison to those of CBT-TAU. If so, this would justify further large-scale evaluations and dissemination efforts.

# STUDY DESIGN

The study is a cost-effectiveness study, designed as a parallel-group randomized controlled trial (N = 156), in which participants are randomly allocated to either bCBT (n=78) or CBT-TAU (n=78). Participants are recruited in Dutch specialized mental healthcare centers (GGZInGeest, GGZAltrecht or GGZ Oost Brabant), large scale mental health service organizations, respectively in the Amsterdam, Utrecht and Noord-Brabant region.

# Measurements are taken at four fixed 6-10-week intervals (depending on the type of anxiety treatment); at baseline (T0), week 6-10, week 12-20 and at weeks 64-72 (52 weeks follow-up) (T1 – T3). The recruitment of participants aims to start April 2015 (first patient in; after Medical Ethics (METC) consent) and the inclusion ends at December 2016, and the follow up at December 2017. For detailed information on the sample size and power calculation, see the study protocol.

**Figure 1 Flowchart for anxiety**

EXCLUSION

bCBT (N=78)

Duration: 12-20 weeks

CBTAU (N=78)

Duration: 12-20 weeks

WEEK 64-72 (T3)

WEEK 12-20 (T2)

WEEK 6-10 (T1)

WEEK 1: BASELINE (T0)

RANDOMIZATION (N=156)

ASSESSMENT OF ELIGIBILITY

ENROLLMENT

# STUDY POPULATION

## Population (base)

## The study targets adults with a current severe anxiety disorder (panic disorder with or without agoraphobia, social phobia, and generalized anxiety disorder), who were referred to and enrolled in an outpatient treatment in specialized mental healthcare center of GGZ Altrecht, GGZ inGeest or GGZ Oost Brabant. The likelihood that the planned number of patients can be recruited from the defined source population is considered high. Recruitment will take place during a time span of 18 months (see flowchart). Recruitment will start at the outpatients departments of GGZingeest ‘De Nieuwe Valerius’, at both outpatient departments of GGZ Altrecht and GGZ Oost-Brabant location Oss. Based on the number of patients that were diagnosed with an anxiety disorder at GGZinGeest last year we would expect around 30 patients per month receiving treatment. We aim to include 8-10 patients per month in the current study. If after 6 months the number of included patients is less than 78 patients, the management of GGZ inGeest has agreed to add two more locations as recruitment sites for this study, yielding a total of approximately 800 patients from which to recruit the intended 156 study participants

## Inclusion criteria

In order to be eligible to participate in this study, a patient must meet all of the following criteria:

- 18 years or older;

- meet the criteria for a severe anxiety disorder (social phobia, panic disorder with or without agoraphobia and generalized anxiety disorder), as determined by a structured interview (SICD-I)

- have sufficient command of the Dutch language, both verbal and written;

- have access to the Internet, an e-mail address and a (tablet) computer;

- are willing to be randomly allocated to on of the two treatments;

- provide signed informed consent.

## Exclusion criteria

A potential subject who meets any of the following criteria will be excluded from participation in this study:

-Primary diagnosis of a bipolar, psychotic or substance abuse disorder and/or acute risk of suicide

-Use of psychotropic medication is not an exclusion criterion

## Sample size calculation

In economic evaluations we are calculating the power to estimate the joint distribution of costs and treatment effects. Subsequently, we need more information for estimating power compared to clinical trials, namely expected costs of treatments, expected covariance of treatment effects/costs, and the maximum willingness to pay for the treatment effect. To incorporate this information, the formula of Glick et al. (2011) can be used. A goal of sample size and power calculation for cost-effectiveness analysis is to identify the likelihood that an experiment will allow us to be confident that a therapy is acceptable or not when we adopt a particular willingness to pay.

For this study a sample size of 156 is based on a formula to estimate the power of a cost-effectiveness analyses. (Glick, 2011)

$$n=\frac{{2(z_{\alpha}+z_{\beta)}}^{2}\left( {sd}^{2}+\left( W^{2}{sd}^{2} \right)-\left( 2W\rho{sd}_{c}{sd}_{q} \right) \right)}{\left( WE-C \right)^{2}}$$

$$n=sample size/group$$

$$z_{\alpha}=z statistics for alpha$$

$$z_{\beta}=z statistics for Beta$$

$${sd}_{c}=Expected standard deviation costs$$

$${sd}_{e}=Expected standard deviation effects$$

$$W=Willingness to pay$$

$$C=Expected differences in costs$$

$$E=Expected differences in effects$$

$$\rho=correlation between differences in costs and effects$$

($z_{\alpha}=1,96$, $z_{\beta}=0,84$,${sd}_{c}=800$, ${sd}_{e}=0,02$, $W=80,000, C=832,E=0,02 \rho=0,1)$

Following the formula, we may expect that the point estimate for the cost-effectiveness ratio will be around 40,000 per quality- adjusted life year (QALY), using only a small increase in quality of life and lower costs for blended eHealth, we want to design an experiment that will provide an 80% chance (i.e., power) to be 95% confident that the therapy is good value when we are willing to pay at most 80,000 per QALY. In the Netherlands, a costs-effectiveness ratio between 20.000 and 80.000 Euro per QALY is assumed acceptable.

Glick, H.A. (2011). Sample size and power for cost-effectiveness analysis (part 1). Pharmacoeconomics, 29 (3), 189-198.

**5. TREATMENT OF SUBJECTS**

Treatments in both groups are based on CBT and exposure with response prevention (ERP) protocols for face-to-face treatment of anxiety disorders according to the Dutch multidisciplinary treatment guidelines for anxiety disorders (panic disorder with or without agoraphobia, social phobia, and generalized anxiety). CBT and ERP are the most recommended treatments for anxiety, according to these guidelines (van Balkom et al, 2013).

The protocols comprise psycho-education (explanation of the treatment rationale and the general procedures in CBT treatment), cognitive therapy (examining relation between thoughts, emotions and behavior), a combination of exposure in vivo with response prevention (ERP; exposure to feared situations combined with prevention of avoidance behaviour and challenging accompanying catastrophic expectations, followed by cognitive restructuring). Depending on the type of disorder all of these components receive more or less attention in the therapy. In case of comorbidity (ie. depression) or when the initial CBT response is insufficient, psychotropic drugs are added; including Serotonin reuptake inhibitors (SSRI's) or as a next step serotonergic Tricyclic Antidepressants. The last part of the protocol will provide information on relapse prevention (identifying and adopting techniques/strategies to prevent depressive symptoms to re-occur). In accordance with standard treatment procedures parallel treatment (such as medication and/or social skills training) is allowed in both conditions, if the practitioner deems this warranted.

### CBT as usual (CBTAU)

In the CBT-TAU condition, patients receive on average 20 45-minutes sessions of face-to-face CBT, spread out over 12-20 weeks (depending on the type of anxiety disorder).

**Blended CBT (bCBT)**

In the bCBT group, patients receive 6-10 face-to-face sessions and 6-10 internet sessions, which will be delivered over a period of 12-20 weeks. Treatment starts and ends with a face-to-face session. The online sessions are delivered through a secured web-based online treatment platform (Minddistrict; www.minddistrict.com). Patients access this platform with a personalized login. The website offers information that repeats and extends the contents of the face-to-face sessions. In addition, patients use the website to complete homework exercises, such as monitoring their activities, feelings, thoughts and behavior. The first online session focuses on working with the online platform. The therapist monitors patients’ online progress and provides weekly feedback prior to the next face-to-face session. Face-to-face sessions will be audio-recorded and checked, following the same procedures as in the CBT-TAU condition. Online sessions are recorded in the online treatment platform database. On completion of treatment, patients can still access the online treatment platform to re-read information and look up homework exercises, such as the relapse prevention plan.

The same medication regimes will be administered for both conditions throughout the study. Pharmacotherapy falls under the responsibility of a psychiatrist and is provided independently of the current study.

# Investigational product/treatment

The software platform serving the blended treatment (Minddisstrict) is not a medical device, based on the 2007/47/EG guideline of the European Parliament, The Wet Medische Hulpmiddelen (Law Medical Aids) (Date of validity 15-09-2013) and the flow chart which was developed by the Nictiz, based on the MEDDEV documentation for medical aids^[[1]](#footnote-1)^. The online part of the intervention can be seen as a digital self-help workbook. The website functions as well a secure environment in which the therapist and the patient can communicate through encoded transmission channels (https).

**Privacy and security information softwareplatform: Minddistrict (www. Minddsitrict.nl)**

**Minddistrict security policy, 08102013**

**Introduction**

This document describes the measures taken by Minddistrict to ensure proper levels of service and security.

**Standards**

Minddistrict’s security policy is based on IS027001 and NEN7510 guidelines

**Data service provider**

Intermax is the Minddistrict ISO 27001 certified data service provider. ISO27001 is the basis for the series of NEN 751x norms. Intermax provides services to several Dutch hospitals and other medical institutions.

Intermax owns and administers several [VMware](http://vmware.com/) clusters, and provides virtual machines to its customers. Intermax takes care of Internet and power connections, networking and firewalling.

Servers are located accross the Netherlands only. Minddistrict is not Patriot act liable.

The virtualization technologies provided by VMware and Intermax allow Minddistrict to scale its operations. Minddistrict procures several virtual machines from Intermax. The virtual machines are Ubuntu/Linux servers.

**Audits**

General scan and penetration tests are performed by Madison Gurkha, at least once a year

**Datacenter location protection**

Intermax datacenter has implemented high level measurements to prevent unauthorized physical access to server areas, including proximity readers, biometric access control, cameras, digital code locks and security personnel.

Only authorised Intermax staff member and authorised Minddistrict (operations) staff members have access to the server space.

Intermax keeps logs of staff entering the server spaces.

**Office location protection**

Minddistrict offices are protected with personal access cards.

**Data protection**

All user data is stored in and confined to Intermax data centers.

No user data is stored anywhere on servers in Minddistrict offices.

**Transport security**

User data is transported using SSL encryption.

Maintenance operations data is transported over a VPN connection.

**Password policy**

Users determine their own passwords.

We apply a severe regime based on 12 characters including 1 special character.

Lost password are handled via a link sent via e-mail.

Passwords are stored in the database in encrypted form, not in plain text.

Systems interface security

Third party systems are connected over a VPN connection.

**Authorisation policy**

In Minddistrict organisation only two roles have access to user data: Minddistrict application managers (2 persons) and deployment managers (1 person).

Access is controlled by NDAs and by keeping access logs.

In the application patients can only access their own data. Professionals can have one or more roles: secretary, therapist, app manager, and analyst. Therapists can see data of their clients, and data of other clients, only after warning and with logging. Managers have access to patients and therapists, but not to individual patient data. Analyst have access (read only) to all data when experting the data.

**Single Sign On**

Minddistrict customers can setup a SSO connection.

Several protocols are supported: delegated login based on encryption and SAML.

**Login session time out**

After 60 minutes a session expires.

**Mobile data storage devices**

The use of mobile data storage devices such as USB and external harddrives is limited to a minimum. When used, data is encrypted.

**Development and production**

Minddistrict operations keeps a strict separation between development, testing, staging and production environments. The production architecture looks like this and is explained in the rest of this document.

**Firewall**

On the outer perimeter of the Minddistrict infrastructure, a redundant [Fortigate](http://www.fortinet.com/products/fortigate/) hardware firewall monitors incoming and outgoing traffic. The firewall is instructed to only allow incoming HTTP and HTTPS traffic from the Internet. The firewall can be instructed to block Denial-Of-Service attacks or throttle traffic.

On the servers behind the first firewall, we use the [iptables](http://en.wikipedia.org/wiki/Iptables) software firewalls in order to monitor traffic between the Minddistrict servers.

**Application servers**

The Minddistrict infrastructure consists of multiple application servers over which the computational load is spread in order to keep response times low.

The application servers are identical servers that accept requests and return web pages. The application servers connect to the database cluster to retrieve the data to base the calculation upon. In case of increasing application load, Minddistrict can scale the infrastructure by adding application servers.

**Database cluster**

Minddistrict uses a [ZEO](http://www.zodb.org/documentation/guide/zeo.html) cluster to store the application data. The cluster consists of multiple nodes, in order to be able to fail-over.

The data of each Minddistrict customer is stored separately in the object database; it is not possible to access the data of customer A’s from the application for customer B.

**Static resource servers**

Static resources (video, audio, javascript, CSS and images) are served by servers dedicated to this task. This leads to faster load times of web pages.

**Load balancing**

The load balancer unwraps SSL traffic and routes the traffic to the proper application or static resources server.

**Backups**

Application data is back-upped on an hourly basis. Every night, a full backup is transfered to an off-site backup server. Every three months, an encrypted backup of these three months is transferred to the vault of a notary.

**Application security**

The Minddistrict application is built using [Zope](http://zope.org/) technology. Zope is a framework for building secure web applications, which has been used for more than 10 years and has an outstanding security record.

All information is transferred over SSL connections.

Email is an insecure medium. The Minddistrict application sends emails, but these never contain confidential information. The emails are pointers to the actual message, which lives in the application and is accessible after logging in with a password.

It is not possible to fish for usernames using the lost-password functionality.

**Server security**

In order to provide proper server security, the following best practices are followed:

Minddistrict administrators have SSH access to the production machines. The user accounts don’t have passwords, SSH-key-based login only. The servers are only accessible over a VPN connection.

Root passwords are stored in a file shared between administrators, encrypted using PGP technology.

Operating system software updates are first tested on testing machines, before being rolled out to staging and production environment. The Administrators subscribe to different security bulletins in order to keep up-to-date on security threats.

**Privacy**

Only certain Minddistrict employees are authorized to access the production servers.

Software bugs are never fixed using hotfixes in the production environment. The production database is downloaded to a testing machine, the data is anonymized before bug hunting. After the bug has been fixed, a new release is made an deployed.

**Monitoring**

The health and performance of the Minddistrict servers and applications are monitored using [Nagios](http://nagios.org/) technology on internal servers and externally through [pingdom](http://pingdom.com/).

[Munin](http://munin.org/) is used to collect historic information, based on which Minddistrict Operations can make informed decisions on how to scale the infrastructure.

## Use of co-intervention (if applicable)

## Not applicable to this study

## *.* INVESTIGATIONAL PRODUCT

Not applicable to this study

# NON-INVESTIGATIONAL PRODUCT

Not applicable to this study.

# METHODS

## Study parameters/endpoints

The health-economic analyses combine clinical outcomes with cost estimates. Measures of these primary variables are described in this section.

### Main study parameter/endpoint

#### Clinical outcomes

Primary clinical outcomes are 1) severity of anxiety symptoms measured with the Beck Anxiety Inventory (BAI), 2) quality-adjusted life years (QALY’s), derived from the Euro Quality of Life questionnaire (EQ-5D-3L) and health-related quality of life, tapped by the SF-36 Health Survey.

### The Beck Anxiety Inventory (BAI) (Beck et al., 1988)consists of twenty-one questions about how the subject has been feeling in the last week, expressed as common symptoms of anxiety (such as numbness and tingling, sweating not due to heat, and fear of the worst happening). It is designed for an age range of 17–80 years old. Each question has the same set of four possible answer choices, which are arranged in columns and are answered by marking the appropriate one with a cross. The BAI has a maximum score of 63. For this study, treatment response is defined as a symptom reduction of the baseline BAI symptom severity score of at least 30% and remission a score reduction of at least 30% reduction plus a total score <11.

**The EQ-5D-3L** (EuroQol, 1990; Lamers, 2005) will be administered at every assessment moment (T0-T3) to assess *general well-being*. The questionnaire consists of five questions that tap mobility, self-care, daily activities, pain and mood. Each item has three response categories, ranging from 0 (*no problems*) to 3 (*severe problems*). In addition to this, participants use a VAS scale to rate their health on a scale ranging from 0 (*worst possible health*) to 100 (*best possible health*). The answers on the five questions are combined in a number sequence that corresponds with the five answers, for example 03210. The total number of possible sequences is 3^5^=243. Each sequence stands for a certain health state. On these health states, a value (utility) has been placed (Lamers et al., 2006), which in turn is used to determine the quality-adjusted life years (QALYs). This is done by calculating the QALYs gained between the follow-up periods by weighing the length of time spent in a particular health condition by the utility score (Drummond, 2005).

### The Short Form Health Survey (SF-36) will be used to assess *health-related quality of life* (Aaronson et al., 1998; Bech et al., 2003; Ware, 2000). This questionnaire consists of 36 questions that are scored on a 8 multi-item scales, which assess physical functioning, role limitations caused by physical health problems, bodily pain, general health perceptions, vitality, social functioning, role limitations caused by emotional problems and general mental health. The raw scores are converted to a scale ranging from 0 to 100 with higher scores being indicative of better levels of functioning. SF-36 scores will provide a second source to determine QALYS, through the application of the Brazier algorithm (Brazier et al., 2002).

Secondary study parameters/endpoints (if applicable).

**Cost outcomes**
Cost estimates include 1) the costs of offering the treatments and 2) Patients’ out-of-pocket costs (non-medical), such as the costs of travelling to the health services and the patients’ time costs of travelling, waiting and receiving treatment, which are determined with the standard cost prices as listed in the pertinent Dutch guideline for economic evaluation (Tan et al., 2012). Furthermore, 3) Costs stemming from health care uptake, including costs of medication, and 4) Costs stemming from productivity losses due to absenteeism and lesser efficiency while at work (presenteeism) are assessed with *The Trimbos/iMTA questionnaire for Costs associated with Psychiatric Illness* (TiC-P; Hakkaart – van Roijen et al., 2002.

### TiC-P is the most widely used health service receipt interview for economic evaluations in the Netherlands. The TiC-P consists of 46 questions, divided over two parts. Part 1 entails the health care uptake at relevant health care providers in the past 4 weeks, such as medication intake, and the number of contacts within the mental health care setting, with the GP and with other medical specialists. To determine the costs associated with these contacts, the care consumption is multiplied by the cost price described in the before mentioned guideline. Part 2 of the TiC-P entails loss of productivity in the past 4 weeks. This is measured by enquiring about the number of days absent from work and the number of days with reduced efficiency due to feeling ill. The costs of productivity losses will be based on the gender and age specific friction costs, as outlined in the Dutch guideline for costing (Hakkaart van Roijen L, 2010).

### Other study parameters (if applicable)

### To further evaluate bCBT compared to CBT-TAU, a number of explorative measures are administered.

The **BDI** (Beck Depression Inventory) (Beck et al., 1961) is a 21-question multiple choice self report inventory of the most widely used instruments for measuring the severity of depression and assesses presence and severity of depressive symptoms.

**The BSI** (Brief Symptom Inventory) (Derogatis and Melisaratos, 1983)is a 53-item, self-report symptom inventory designed to evaluate general psychopathology. It is a brief form of the SCL-90 and is designed to provide a multidimensional symptom measurement in about 10 minutes.

**PDSS** (Panic Disorder Severity Scale (PDSS) (Shear et al., 1997) is one tool that can be used to assess the severity of panic attacks. The scale is fairly simple. There are 7 questions, each one with 5 answers that are scored 0 to 4. That leads to 28 total points possible with this scale. Ascore over 9 is considered important enough to be of clinical significance..

**LSAS** (Liebowitz Social Anxiety Scale) originally developed by Liebowitz (1987) is a short questionnaire to assess the range of social interaction and performance situations feared by a patient in order to assist in the diagnosis of social anxiety disorder. The scale features 24 items, 13 relating to performance anxiety and 11 concerning social situations and has been validated as a self-report scale (Rytwinski et al., 2009)

The **PSWQ** (Penn State Worry Questionnaire) (Meyer et al., 1990)is a self-report measure to assess pathological worry in GAD patients. By adding up the value (five-point scale, range 1–5) of all 16 items (e.g., “I’m always worrying about something“); leading to a total score from 16 to 80.

The **(WSAS)** Work and Social Adjustment Scale (Mundt et al., 2002)is a 5-item patient self-report measure, which assesses the impact of a person’s mental health difficulties on their ability to function in terms of work, home management, social leisure, private leisure and personal or family relationships. The WSAS is used for all patients with depression or anxiety as well as phobic disorders.

The five item version of **The Pearlin Mastery Scale** (Pearlin and Schooler, 1978) is administered at each assessment moment (T0-T3) to assess changes in *locus of control*. Locus of control could potentially mediate treatment effect and facilitate relapse prevention. The questionnaire consists of five questions, which are scored on a five-point Likert-scale, ranging from 1 (*totally disagree*) to 5 (*totally agree*). The total score ranges from 5 to 30, with higher scores being indicative of a higher level of experienced control.

The 12-item version of **the Work Alliance Inventory** (WAV-12; (Andrusyna et al., 2001) is used to let patients rate the *quality of the work alliance* between patient and therapist at T1 (week 10). The questionnaire is administered to investigate whether the blended treatment for has an effect on the quality of the work alliance.

The questionnaire consists of 12 items, which are scored on a five-point Likert-scale, ranging from 1 (seldom or never) to 5 (always). The raw scores range from 12 to 60, with higher scores being indicative of a better alliance between therapist and patient.

**Demographic characteristics** such as age, sex, education, employment and marital status will be collected with a general demographic questionnaire. Additional questions are asked concerning **clinical anxiety characteristics** such as age of onset, number of months depressed in past 4 years, duration of current episode, medical illnesses and treatment status. In addition, patients indicate their **treatment preference** (BCBT / CBT-TAU). Finally, participants are asked about their **computer use**: number of hours spent behind a computer and reasons for use.

#### Patient evaluations

**The Client Satisfaction Questionnaire-8** (CSQ-8; (Larsen et al., 1979) will be administered at week 30 (T3). The CSQ consists of 8 questions with item-specific response categories. The total score ranges from 8 to 32, with higher scores being indicative of higher *levels of client satisfaction*.

#### The System Usability Scale (SUS, Brooke, 1996) will be administered at week 20 amongst the participants randomized to the bCBT group. The SUS consists of 10 questions with 5 response options, ranging from 0 (strongly disagree) to 4 (strongly agree). The total scores are converted to a scale ranging from 0 to 100. Higher scores are indicative of higher *usability of the online platform* that is used for the Internet sessions in the blended therapy.

#### Process data

Data for process analyses are obtained from the administration of the participating mental health care institutions and through usage statistics of the online platform. We will consider the following aspects:

**Recruitment**: how much time is required for the recruitment of patients?

**Treatment adherence**: What is the percentage of dropout during therapy, when do patients drop out (number of completed sessions)? What is the reason for treatment dropout? Number of face-to-face contacts and number of cancellations. Do patients complete homework exercises?

**Time investment**: By both the patient and the therapist.

**Table 1: Overview of measurements for anxiety.**

| **Questionnaire** | **Baseline**  **Start treatment**  **(T0)** | **Week 6-10**  **Mid-treatment**  **(T1)** | **Week 12-20**  **End treatment**  **(T2)** | **Week 64-72**  **Follow-uptreatment**  **(T3)** |
| --- | --- | --- | --- | --- |
| **Primary outcomes** |  |  |  |  |
| BAI (anxiety severity) | x | x | x | x |
| SCID-I full diagnostic interview | x |  |  | x |
| EQ-5D (general well-being) | x | x | x | x |
| SF-36 (health-related quality of life) | x | x | x | x |
| TiC-P (health-care utilization) | x | x | x | x |
|  |  |  |  |  |
| **Secondary outcomes** |  |  |  |  |
| PDDS (panic disorder, if applicable) | x | x | x | x |
| LSAS (social anxiety, if applicable) | x | x | x | x |
| PSWQ (Generalized Anxiety Disorder, if applicable) | x | x | x | x |
| BDI (depression) | x | x | x | x |
| WSAS (work and social adjustment) | x | x | x | x |
| BSI (general psychopathology) | x | x | x | x |
| CSQ (satisfaction)  Mastery Scale (locus of control)  WAI (therapeutic alliance)  SUS (system usability, bCBT only) | x | x  x | x  x | x  x |

## Randomisation, blinding and treatment allocation

Participants will be randomly assigned to either bCBT or CBT-TAU by an independent researcher, on the basis of a computer-generated block randomization table. Group allocation cannot be blinded to patients and therapists. However, outcome assessors (i.e., those conducting the SCID-I diagnostic interviews at T0 to T3) will be blinded to the allocation, in accordance to the CONSORT guidelines (Moher et al., 2010; Schulz et al., 2012).

## Study procedures

Patients will be recruited within outpatient Anxiety Disorder departments of the participating Mental Health Care site(s) of GGZingeest, GGZ Altrecht and GGZ Oost-Brabant in the Netherlands. During their intake interview, all new patients indicate whether they are willing to participate in a study. All patients with a primary diagnosis of a severe anxiety disorder (panic disorder with or without agoraphobia, social phobia, and generalized anxiety disorder) who are willing to participate will be approached by the researchers by telephone and asked to participate in the study. Participants then receive a letter containing information on the study and the informed consent form (including a return envelope).

After a week, the researchers will contact the patient again. When the patient is willing to participate, a trained researcher then screens the patients with respect to the inclusion and exclusion criteria based on the SCID-I diagnosis and the patient’s demographic characteristics, such as age.

Patients who are not eligible to participate will be notified and remitted to regular treatment trajectories within the participating specialized mental health care center(s).

If patients are eligible to participate, written informed consent will be obtained. Subsequently, patients receive an email containing an invitation to fill in the online questionnaires. When the baseline measures are completed, patients are randomized to either bCBT or CBT-TAU. Treatment duration is 12-20 weeks in the bCBT group and 12-20 weeks in the CBT-TAU group. When necessary, additional treatments are allowed for all patients at any point of time during the study.

After 6-10, 12-20 and after 64-72 weeks (52 weeks follow-up), the patient is asked to complete a number of measures (see Table 1), which consist of a diagnostic interview (SCID-I and a set of online questionnaires).

At week 64-72 (52 weeks follow-up) the full SCID-I interview is administered to assess whether the anxiety disorder is present and to determine co-morbid disorders. The SCID-I can be administered via the telephone and face-to-face at a the mental health care location, depending on patient preference. A full SCID-I interview takes 60 minutes at most. Administering sections on anxiety disorders takes about 10 minutes.
The self-report questionnaires are administered online. Patients receive an email with a personal link to their questionnaires and have one week to fill them in. The questionnaires take 30 to 45 minutes to complete.
If the measurements that are administered in the study (during and after treatment) indicate signs of relapse or suicidal ideation, the investigator will contact the therapist immediately.

## Withdrawal of individual subjects

Subjects can leave the study at any time for any reason if they wish to do so without any consequences. The investigator can decide to withdraw a subject from the study for urgent medical reasons.

### Specific criteria or withdrawal (if applicable)

### Patients are informed that participation in the study is voluntary and they have the right to withdraw consent or discontinue participation at any time, with or without stating a reason for withdrawal. Because it is not always recommended to end treatment abruptly, the patient is advised to discuss ending treatment with his or her therapist first. The decision to end study participation will not have negative effects on the further treatment, the care, and the attention each patient has a right to within GGZ Altrecht,GGZ ingest and GGZ Oost-Brabant

## Replacement of individual subjects after withdrawal

Not applicable.

## Follow-up of subjects withdrawn from treatment

Participants who decide to withdraw from treatment are asked to provide the reason for withdrawal and to fill in the post-test measurement. Participants will not be pressured or forced to comply with this request.

## Premature termination of the study

## Not applicable.

# SAFETY REPORTING

## Section 10 WMO event

In accordance to section 10, subsection 1, of the WMO, the investigator will inform the subjects and the reviewing accredited METC if anything occurs, on the basis of which it appears that the disadvantages of participation may be significantly greater than was foreseen in the research proposal. The study will be suspended pending further review by the accredited METC, except insofar as suspension would jeopardize the subjects’ health. The investigator will take care that all subjects are kept informed.

## AEs, SAEs and SUSARs

### Adverse events (AEs)

Adverse events are defined as any undesirable experience occurring to a subject during the study, whether or not considered related to [the investigational product / the experimental intervention]. All adverse events reported spontaneously by the subject or observed by the investiga­tor or his staff will be recorded.

### Serious adverse events (SAEs)

A serious adverse event is any untoward medical occurrence or effect that at any dose:

- results in death;
- is life threatening (at the time of the event);
- requires hospitalisation or prolongation of existing inpatients’ hospitalisation;
- results in persistent or significant disability or incapacity;
- is a congenital anomaly or birth defect;
- Any other important medical event that may not result in death, be life threatening, or require hospitalization, may be considered a serious adverse experience when, based upon appropriate medical judgement, the event may jeopardize the subject or may require an intervention to prevent one of the outcomes listed above.

Mental Health Care Institutions GGZ Altrecht, GGZ inGeest and GGZ Oost-Brabant already have quality guidelines instated, which include safety protocols (e.g., the Quality Law for Health Institutions– de Kwaliteitswet Zorginstellingen). These will be adhered to in the current study, as treatment will be delivered within the regular treatment procedures. The participating centers will notify the primary investigator (Jeroen Koning) of possible SAEs within 24 hours.

0

The sponsor will report the SAEs through the web portal *ToetsingOnline* to the accredited METC that approved the protocol, within 15 days after the sponsor has first knowledge of the serious adverse events.

SAEs that result in death or are life threatening should be reported expedited. The expedited reporting will occur not later than 7 days after the responsible investigator has first knowledge of the adverse event. This is for a preliminary report with another 8 days for completion of the report.

*< If certain SAEs do not require immediate reporting, please specify these SAEs as well as the frequency of reporting of these SAEs in line listings.*>

### Suspected unexpected serious adverse reactions (SUSARs)

<This c*hapter is only applicable for studies with an investigational medicinal product>*

Adverse reactions are all untoward and unintended responses to an investigational product related to any dose administered.

Unexpected adverse reactions are SUSARs if the following three conditions are met:

1. the event must be serious (see chapter 9.2.2);
2. there must be a certain degree of probability that the event is a harmful and an undesirable reaction to the medicinal product under investigation, regardless of the administered dose;
3. the adverse reaction must be unexpected, that is to say, the nature and severity of the adverse reaction are not in agreement with the product information as recorded in:

- Summary of Product Characteristics (SPC) for an authorised medicinal product;
- Investigator’s Brochure for an unauthorised medicinal product.

The sponsor will report expedited the following SUSARs through the web portal *ToetsingOnline* to the METC:

- SUSARs that have arisen in the clinical trial that was assessed by the METC;
- SUSARs that have arisen in other clinical trials of the same sponsor and with the same medicinal product, and that could have consequences for the safety of the subjects involved in the clinical trial that was assessed by the METC.

The remaining SUSARs are recorded in an overview list (line-listing) that will be submitted once every half year to the METC. This line-listing provides an overview of all SUSARs from the study medicine, accompanied by a brief report highlighting the main points of concern.

The expedited reporting of SUSARs through the web portal ToetsingOnline is sufficient as notification to the competent authority.

The sponsor will report expedited all SUSARs to the competent authorities in other Member States, according to the requirements of the Member States.

The expedited reporting will occur not later than 15 days after the sponsor has first knowledge of the adverse reactions. For fatal or life threatening cases the term will be maximal 7 days for a preliminary report with another 8 days for completion of the report.

*< For multicentre studies the responsibilities of investigators in participating centres as well as of the coordinating investigator should be clearly defined>*

<*Please describe also the method of breaking the code for SUSAR reporting*.>

## Annual safety report

<This c*hapter is only applicable for studies with an investigational medicinal product>*

< *The annual safety report may be combined with the annual progress report (see chapter 12.4)*.>

In addition to the expedited reporting of SUSARs, the sponsor will submit, once a year throughout the clinical trial, a safety report to the accredited METC, competent authority, and competent authorities of the concerned Member States.

This safety report consists of:

- a list of all suspected (unexpected or expected) serious adverse reactions, along with an aggregated summary table of all reported serious adverse reactions, ordered by organ system, per study;
- a report concerning the safety of the subjects, consisting of a complete safety analysis and an evaluation of the balance between the efficacy and the harmfulness of the medicine under investigation.

## Follow-up of adverse events

All AEs will be followed until they have abated, or until a stable situation has been reached. Depending on the event, follow up may require additional tests or medical procedures as indicated, and/or referral to the general physician or a medical specialist.

SAEs need to be reported till end of study within the Netherlands, as defined in the protocol

## [Data Safety Monitoring Board (DSMB) / Safety Committee]

<*In case a DSMB is established to perform ongoing safety surveillance and to perform interim analyses on the safety data, this committee should be an independent committee. The composition of the DSMB should be described and it should be clear that each member has no conflict of interest with the sponsor of the study*.

*The task and responsibility of the DSMB should be described (see chapter 10.4 for interim analyses either for safety or for futility or positive efficacy)* >

<*Criteria on which the DSMB may decide to terminate the trial prematurely should be clearly defined before the trial has started.*>

The advice(s) of the DSMB will only be sent to the sponsor of the study. Should the sponsor decide not to fully implement the advice of the DSMB, the sponsor will send the advice to the reviewing METC, including a note to substantiate why (part of) the advice of the DSMB will not be followed.

*< In case a DSMB is not needed, but some safety review is deemed appropriate, information on this safety committee should be given here. Information should be provided on the composition of the committee and (in)dependence of the members, the reason to establish this committee, type of data that will be reviewed and moment of review, possible measures to be taken>*

# STATISTICAL ANALYSIS

## Primary analyses

A cost-effectiveness analysis (CEA) and cost-utility analysis (CUA) will be conducted from the societal perspective. In addition, a budget impact analysis (BIA) will be based on a health-economic modeling study in agreement to Mauskopf’s recommendations (2007), i.e. from the perspective of the public purse, the health care insurer and the service-provider.

**CEA/CUA**

Costs will be assessed at pre-, mid-, post-treatment and at one-year follow up. As the TIC-P cost date covers a period of three months, all costs will be extrapolated to a 12 months period assuming a stability of costs during the time frame. T-tests will test the change for each cost domain. As cost data will not be normally distributed, p-values will be estimated using a multilevel model (with repeated measurements to control for dependency between the measurements) after which a bootstrap analysis on the parameters is performed (10,000 replications, to generate a reliable cost estimate [23]. An incremental cost-effectiveness ratio (costs per case response or remission) and will be calculated (ICER =(mean costs blended eHealth treatment-mean costs TAU)/(mean bCBT -mean CBT-TAU). The mean costs, including all medical and non medical costs, of the patients in the blended eHealth condition will be subtracted from the mean costs of the patients in the TAU condition. This difference will be then be divided with the subtracted effects (case of response or remission on the BAI). This procedure will be bootstrapped 10,000 times, generating an estimation of the blended eHealth treatment groups’ incremental costs in relation to their incremental health benefit. Additionally an incremental cost-utility ratio (costs per QALY) will be calculated; this procedure is identical to the cost-effectiveness ratio with the exception that instead of a case of response or remission, an additional quality adjusted life year is calculated (QALY). Finally, to test the robustness of the results, we will conduct a sensitivity analyses, to investigate how sensitive the ICERs will be to changes of cost estimates (for example difference in costs per iCBT contact, type of psychologists and number of sessions). For decision-making purposes, the ICER acceptability curve will be plotted for various willingness-to-pay (WTP) ceilings, which helps making judgments about whether the blended intervention offers good value for money, relative to treatment as usual. One-way sensitivity analyses directed at uncertainty in the main cost drivers will be performed to gauge the robustness of our findings across a range of likely values of those parameters.

**BIA**

To assess how health care budgets are changed by offering blended CBT for depression compared to CBT as usual, a *budget impact analysis* (BIA) will be conducted as outlined in Mauskopf et al. (2007). The BIA will include 1) the perspective of the public purse (in Dutch: Budgettair Kader Zorg), and 2) the perspective of the health care insurer and health care service provider. For each perspective, we consider costs when 10%, 20%, 30% and 100% of the target group receive bCBT. These scenarios will be compared with the base-case scenario, reflecting current care, where 0% of the target group is offered bCBT. When taking the societal perspective, we will consider the costs of offering the health care interventions (also the interventions offered in routine medical care for this particular target group), patients’ out of pocket costs, and costs stemming from productivity losses. Treatment costs will be based on the full economic cost prices (standard cost prices as reports in the Dutch Costing Manual). Productivity losses will be valued using the average gender and age specific productivity levels in the Dutch working population. When taking the perspectives of the public purse (BKZ) and the health insurance companies’ perspective, the focus will be restricted solely to the direct medical costs. For this, the average tariffs of the Dutch Health Authority will be used (NZa). The Budget Impact Analysis (BIA) will be conducted using a health economic (Markov cohort) simulation model.

## Secondary study parameter(s)

Outcomes on continuous clinical outcome variables, such as depressive symptoms, at T1, T2 and T3 (week 6-10, 12-20, and 64-72) are estimated for descriptive purposes through mixed-model analyses (MM), with participants as random effects, and time (T1-T3), group (blended vs. face-to-face treatment) and time x group as fixed effects, with baseline scores as a single covariate. To assess the magnitude of treatment effects, Cohen’s *d* effect sizes (Cohen, 1988) for each time point are calculated by dividing MM parameter estimates of fixed effects at each post-treatment assessment by the pooled standard deviation of outcome measurements at baseline (T0).

## Interim analysis (if applicable)

# Not applicable ETHICAL CONSIDERATIONS

## Regulation statement

The study will be conducted according to the principles of the Declaration of Helsinki (seventh version, 2013) and in accordance with the Medical Research Involving Human Subjects Act (WMO).

## Recruitment and consent

Patients will be recruited within the Anxiety Disorder Department of the participating Mental Health Care site(s) of GGZ inGeest, GGZ Altrecht and GGZ Oost-Brabant in the Netherlands. All new patients indicate during their intake interview whether they are willing to participate in a study. All patients with a primary diagnosis of a severe anxiety disorder (panic disorder with or without agoraphobia, social phobia, and generalized anxiety disorder) who are willing to participate who have agreed to be approached are contacted by the researchers by telephone and asked to participate in the study. Participants then receive a letter containing information on the study and the informed consent form (including a return envelope).

After a week, the researchers will contact the patient again. When the patient is willing to participate, a trained researcher then screens the patients with respect to the inclusion and exclusion criteria based on the SCID-I diagnosis and the patient’s demographic characteristics, such-as age. TheSCID-I interview is performed over the telephone, unless the patient prefers to do this face-to-face at a GGZ inGeest, GGZ Altrecht or GGZ Oost-Brabant location.

Patients who are not eligible to participate will be notified and remitted to regular treatment trajectories within the participating specialized mental health care center(s). In case of acute threat of suicide the principal investigator will inform the professional responsible for treatment immediately via telephone and e-mail.

If patients are eligible to participate, written informed consent will be obtained via mail.

## Objection by minors or incapacitated subjects (if applicable)

## The blended CBT treatment that will be provided in this trial does not add risks to the CBT treatment as usual. The questionnaires can be considered a burden.

## Benefits and risks assessment, group relatedness

An insurance exemption is requested from the METC VUmc. No risks are associated with participation in the study.

## Compensation for injury

Not applicable.

The sponsor/investigator has a liability insurance which is in accordance with article 7, subsection 9 of the WMO.

The sponsor (also) has an insurance which is in accordance with the legal requirements in the Netherlands (Article 7 WMO and the Measure regarding Compulsory Insurance for Clinical Research in Humans of 23th June 2003). This insurance provides cover for damage to research subjects through injury or death caused by the study.

1. € 450.000,-- (i.e. four hundred and fifty thousand Euro) for death or injury for each subject who participates in the Research;
2. € 3.500.000,-- (i.e. three million five hundred thousand Euro) for death or injury for all subjects who participate in the Research;
3. € 5.000.000,-- (i.e. five million Euro) for the total damage incurred by the organisation for all damage disclosed by scientific research for the Sponsor as ‘verrichter’ in the meaning of said Act in each year of insurance coverage.

The insurance applies to the damage that becomes apparent during the study or within 4 years after the end of the study.

## Incentives (if applicable)

<*Please describe any special incentives, compensation or treatment that subjects will receive through participation in the study*.>

# ADMINISTRATIVE ASPECTS, MONITORING AND PUBLICATION

## Handling and storage of data and documents

During the study personal patient data (such as contact information, demographic variables and information concerning inclusion) will be stored in an administrative database at a GGZ inGeest server, which is secured with a password. The data will be collected as part of the regular intake procedure at GGZ inGeest, GGZ Altrecht and GGZ Oost-Brabant and is accessible to the partaking investigators (Jeroen Koning and Geke Romijn). .

The data that are collected during the study (questionnaires at baseline, week 6-10, 12-20 and week 64-72), will be stored in secure databases on the secure servers of inGeest, GGZ Altrecht and GGZ Oost Brabant. The information that is needed for the current study will be transferred by a GGZ inGeest, GGZ Altrecht and GGZ Oost-Brabant data-manager to a secure database at the VU. The databases will be secured with a password that is only known to the principal investigators.

Participants will be registered with a seven-figure code with which they can be identified in the database. The first three numbers of the code refers to the center in which the participants receive treatment. The last three numbers refer to the patient. The link between the personal data and the number is kept in a password secured patient identification log-document that is only accessible to one investigator.

The data that is collected via the web-modules of the blended treatment will be stored at a MindDistrict server. MindDistrict operates according to the ISO 27001 norm (International Organization for Standardization, 2005) and is currently in the process of becoming certified according to the ISO 27001 norm. The hosting party is ISO 27001 certified. Information that will be collected via the online questionnaires is collected with NetQuestionnaire. The information acquired via NetQuestionnaire is stored at servers at GGZ inGeest.
All online data will be sent via HTTPS with a SSL-certificate (AES-256 and SHA-1, RSA (2048 bits)). HTTPS entails an extension to the HTTP-protocol (the protocol for communication between a webclient and a webserver) with which the information is encrypted during transfer, so an outsider or intruder should not be possible to access the information.

## Monitoring and Quality Assurance

The study will be conducted according to the recommendations of the quality handbook of EMGO+ (<http://www.emgo.nl/kc/>). The investigators have received training in following the rules and regulations that are stated in the handbook. The quality of data collection is further strengthened by the support of the data-management staff of GGZ inGeest. The quality of the study procedures are not otherwise monitored, but there is a possibility that the study will be randomly selected for an independent audit of EMGO+.

## Amendments

Amendments are changes made to the research after a favorable opinion by the accredited METC has been given. All amendments will be notified to the METC that gave a favorable opinion.

Non-substantial amendments will not be notified to the accredited METC and the competent authority, but will be recorded and filed by the sponsor.

<*The following text is applicable for studies with an investigational medicinal product.*>

A ‘substantial amendment’ is defined as an amendment to the terms of the METC application, or to the protocol or any other supporting documentation, that is likely to affect to a significant degree:

- the safety or physical or mental integrity of the subjects of the trial;
- the scientific value of the trial;
- the conduct or management of the trial; or
- the quality or safety of any intervention used in the trial.

All substantial amendments will be notified to the METC and to the competent authority.

Non-substantial amendments will not be notified to the accredited METC and the competent authority, but will be recorded and filed by the sponsor.

< *Examples of non-substantial amendments are typing errors and administrative changes like changes in names, telephone numbers and other contact details of involved persons mentioned in the submitted study documentation*.>

## Annual progress report

The sponsor/investigator will submit a summary of the progress of the trial to the accredited METC once a year. Information will be provided on the date of inclusion of the first subject, numbers of subjects included and numbers of subjects that have completed the trial, serious adverse events/ serious adverse reactions, other problems, and amendments.

## End of study report

<*The following text is applicable for studies without an investigational medicinal product*.>

The investigator will notify the accredited METC of the end of the study within a period of 8 weeks. The end of the study is defined as the last patient’s last visit.

In case the study is ended prematurely, the investigator will notify the accredited METC within 15 days, including the reasons for the premature termination.

 Within one year after the end of the study, the investigator/sponsor will submit a final study report with the results of the study, including any publications/abstracts of the study, to the accredited METC.

<*The following text is applicable for studies with an investigational medicinal product.*>

The sponsor will notify the accredited METC and the competent authority of the end of the study within a period of 90 days. The end of the study is defined as the last patient’s last visit.

In case the study is ended prematurely, the sponsor will notify the accredited METC and the competent authority within 15 days, including the reasons for the premature termination.

 Within one year after the end of the study, the investigator/sponsor will submit a final study report with the results of the study, including any publications/abstracts of the study, to the accredited METC and the Competent Authority.

<*In case the final study report will not be available within one year, another term should be defined including the reasons.*>

## Public disclosure and publication policy

# The results will be described in a report to ZonMw, the institution that subsidized the current pilot trial. In addition, the results will be published in at least one international peer-reviewed journal

# STRUCTURED RISK ANALYSIS

*<This chapter is applicable for research with any product: medicinal product, food product, medical device or other (as described in chapter 6 and 7)>*

## Potential issues of concern

*< In this final paragraph of the research protocol a structured risk analysis which consists of a number of steps is required. The analysis should result in a comprehensive overall synthesis of the direct risks for the research subjects in this study in chapter 13.2. The risk considerations on the various issues listed below should be supported by up to date information and should be clearly described to allow a thorough review by the METC. For details one may refer to the previous chapters, the Investigator’s Brochure (IB) or a similar document (if applicable), peer reviewed papers in (biomedical/scientific) journals. The issues below are provided to structure your considerations and allows an efficient communication with the METC when questions arise as a result of the review of your research protocol. The remarks per item are provided as a guidance for describing your considerations. Should issues not be applicable, please indicate so.*

*For registered products to be used within the indication and* ***not*** *in combination with other products chapter 13.1 can be skipped; explain in chapter 13.2 why 13.1 is skipped >*

a. Level of knowledge about mechanism of action

b. Previous exposure of human beings with the test product(s) and/or products with a similar biological mechanism

c. Can the primary or secondary mechanism be induced in animals and/or in *ex-vivo* human cell material?

d. Selectivity of the mechanism to target tissue in animals and/or human beings

e. Analysis of potential effect

f. Pharmacokinetic considerations

g. Study population

h. Interaction with other products

i. Predictability of effect

j. Can effects be managed?

## Synthesis

*<should include uncertainties and the unknown and the overall risk:*

*Make clear what measures have been taken to reduce what risks*

*Make clear why in your opinion the remaining risks are acceptable for the subjects participating in the study>*

# REFERENCES

Aaronson, N.K., Muller, M., Cohen, P.D., Essink-Bot, M.L., Fekkes, M., Sanderman, R., Sprangers, M.A., te Velde, A., Verrips, E., 1998. Translation, validation, and norming of the Dutch language version of the SF-36 Health Survey in community and chronic disease populations. Journal of clinical epidemiology 51, 1055-1068.

Andersson, G., Cuijpers, P., Carlbring, P., Riper, H., Hedman, E., 2014. Guided Internet-based vs. face-to-face cognitive behavior therapy for psychiatric and somatic disorders: a systematic review and meta-analysis. World psychiatry : official journal of the World Psychiatric Association 13, 288-295.

Andrews, G., Cuijpers, P., Craske, M.G., McEvoy, P., Titov, N., 2010. Computer therapy for the anxiety and depressive disorders is effective, acceptable and practical health care: a meta-analysis. PloS one 5, e13196.

Andrusyna, T.P., Tang, T.Z., DeRubeis, R.J., Luborsky, L., 2001. The factor structure of the working alliance inventory in cognitive-behavioral therapy. The Journal of psychotherapy practice and research 10, 173-178.

Bakker, P., Jansen, P., 2013. Generalistische Basis GGZ: verwijsmodel en productbeschrijvingen.

Bech, P., Olsen, L.R., Kjoller, M., Rasmussen, N.K., 2003. Measuring well-being rather than the absence of distress symptoms: a comparison of the SF-36 Mental Health subscale and the WHO-Five Well-Being Scale. International journal of methods in psychiatric research 12, 85-91.

Beck, A.T., Epstein, N., Brown, G., Steer, R.A., 1961. "An inventory for measuring depression". . Arch. Gen. Psychiatry 4 561–571.

Beck, A.T., Epstein, N., Brown, G., Steer, R.A., 1988. An inventory for measuring clinical anxiety: psychometric properties. Journal of consulting and clinical psychology 56, 893-897.

Bijl, R.V., de Graaf, R., Hiripi, E., Kessler, R.C., Kohn, R., Offord, D.R., Ustun, T.B., Vicente, B., Vollebergh, W.A., Walters, E.E., Wittchen, H.U., 2003. The prevalence of treated and untreated mental disorders in five countries. Health Aff (Millwood) 22, 122-133.

Brazier, J., Roberts, J., Deverill, M., 2002. The estimation of a preference-based measure of health from the SF-36. Journal of health economics 21, 271-292.

Bruce, S.E., Yonkers, K.A., Otto, M.W., Eisen, J.L., Weisberg, R.B., Pagano, M., Shea, M.T., Keller, M.B., 2005. Influence of psychiatric comorbidity on recovery and recurrence in generalized anxiety disorder, social phobia, and panic disorder: a 12-year prospective study. The American journal of psychiatry 162, 1179-1187.

Cuijpers, P., Marks, I.M., van Straten, A., Cavanagh, K., Gega, L., Andersson, G., 2009. Computer-aided psychotherapy for anxiety disorders: a meta-analytic review. Cognitive behaviour therapy 38, 66-82.

Derogatis, L.R., Melisaratos, N., 1983. The Brief Symptom Inventory: an introductory report. Psychological medicine 13, 595-605.

Drummond, M., .Sculpher, MJ. Torrance, GW. O'Brien, BJ. Stoddart, GL., 2005. Methods for economic evaluation of health care programmes, Third ed. Oxforf University Press, Oxford.

EuroQol, 1990. EuroQol--a new facility for the measurement of health-related quality of life. Health policy (Amsterdam, Netherlands) 16, 199-208.

First, M.B., Spitzer, R.L., Gibbon M., Williams, J. B.W.,, 1997. Structured Clinical Interview for DSM-IV Axis I Disorders, Clinician Version (SCID-CV). . American Psychiatric Press, Inc., Washington, D.C.

First, M.B., Spitzer, R.L., Gibbon M., Williams, J. B.W.,, 2002. Structured Clinical Interview for DSM-IV-TR Axis I Disorders. Biometrics Research, New York State Psychiatric Institute, New York.

Gerhards, S.A., de Graaf, L.E., Jacobs, L.E., Severens, J.L., Huibers, M.J., Arntz, A., Riper, H., Widdershoven, G., Metsemakers, J.F., Evers, S.M., 2010. Economic evaluation of online computerised cognitive-behavioural therapy without support for depression in primary care: randomised trial. The British journal of psychiatry : the journal of mental science 196, 310-318.

GGZNederland, 2010. Zorg op waarde geschat Update Sector rapport ggz 2010. GGZ Nederland, Amersfoort.

Graaf R de, H.M.t., Dorsselear S van., 2010. NEMESIS-2: opzet en eerste resultaten. Trimbos Instituut, Utrecht.

Hakkaart van Roijen L, T.S.S., Bouwmans C.A.M., 2010. Handleiding voor kosten onderzoek, in: CVZ (Ed.). Medical Technology Assessment, Erasmus Universiteit Rotterdam, the Netherlands.

Haug, T., Nordgreen, T., Ost, L.G., Havik, O.E., 2012. Self-help treatment of anxiety disorders: a meta-analysis and meta-regression of effects and potential moderators. Clinical psychology review 32, 425-445.

Hedman, E., El Alaoui, S., Lindefors, N., Andersson, E., Ruck, C., Ghaderi, A., Kaldo, V., Lekander, M., Andersson, G., Ljotsson, B., 2014. Clinical effectiveness and cost-effectiveness of Internet- vs. group-based cognitive behavior therapy for social anxiety disorder: 4-year follow-up of a randomized trial. Behaviour research and therapy 59, 20-29.

Hedman, E., Ljotsson, B., Lindefors, N., 2012. Cognitive behavior therapy via the Internet: a systematic review of applications, clinical efficacy and cost-effectiveness. Expert review of pharmacoeconomics & outcomes research 12, 745-764.

Lamers, L.M., Stalmeier, P.F.M., McDonnell, J., Krabbe, P.F.M., Busschbach van, J.J., 2005. Kwaliteit van leven in economische evaluaties: het Nederlands EQ-5D tarief. Nederlandse Tijdschrift voor Geneeskunde 149, 5.

Larsen, D.L., Attkisson, C.C., Hargreaves, W.A., Nguyen, T.D., 1979. Assessment of client/patient satisfaction: development of a general scale. Evaluation and program planning 2, 197-207.

Lewis, C., Pearce, J., Bisson, J.I., 2012. Efficacy, cost-effectiveness and acceptability of self-help interventions for anxiety disorders: systematic review. The British journal of psychiatry : the journal of mental science 200, 15-21.

Lokkerbol, J., Adema, D., Cuijpers, P., Reynolds, C.F., 3rd, Schulz, R., Weehuizen, R., Smit, F., 2014. Improving the cost-effectiveness of a healthcare system for depressive disorders by implementing telemedicine: a health economic modeling study. The American journal of geriatric psychiatry : official journal of the American Association for Geriatric Psychiatry 22, 253-262.

Mayo-Wilson, E., Montgomery, P., 2013. Media-delivered cognitive behavioural therapy and behavioural therapy (self-help) for anxiety disorders in adults. The Cochrane database of systematic reviews 9, CD005330.

Meyer, T.J., Miller, M.L., Metzger, R.L., Borkovec, T.D., 1990. Development and validation of the Penn State Worry Questionnaire. Behaviour research and therapy 28, 487-495.

Mundt, J.C., Marks, I.M., Shear, M.K., Greist, J.H., 2002. The Work and Social Adjustment Scale: a simple measure of impairment in functioning. The British journal of psychiatry : the journal of mental science 180, 461-464.

Nordgren, L.B., Hedman, E., Etienne, J., Bodin, J., Kadowaki, A., Eriksson, S., Lindkvist, E., Andersson, G., Carlbring, P., 2014. Effectiveness and cost-effectiveness of individually tailored Internet-delivered cognitive behavior therapy for anxiety disorders in a primary care population: a randomized controlled trial. Behaviour research and therapy 59, 1-11.

Pearlin, L.I., Schooler, C., 1978. The structure of coping. Journal of health and social behavior 19, 2-21.

Reger, M.A., Gahm, G.A., 2009. A meta-analysis of the effects of internet- and computer-based cognitive-behavioral treatments for anxiety. Journal of clinical psychology 65, 53-75.

Riper, H., van Ballegooijen, W., Kooistra, L., de Wit, W., Donker, T., 2013. Preventie van Angstaandoeningen via Internet, in: ZonMW (Ed.), Kennissynthese eMental-health: onderzoek dat leidt, technologie die verleidt, en preventie die bereikt en beklijft. ZonMW.

RIVM, 2013. Nationaal Kompas Volksgezondheid, pp. <http://www.nationaalkompas.nl/gezondheid-en-ziekte/ziekten-en-aandoeningen/psychische-stoornissen/angststoornissen/>.

Rytwinski, N.K., Fresco, D.M., Heimberg, R.G., Coles, M.E., Liebowitz, M.R., Cissell, S., Stein, M.B., Hofmann, S.G., 2009. Screening for social anxiety disorder with the self-report version of the Liebowitz Social Anxiety Scale. Depression and anxiety 26, 34-38.

Shear, M.K., Brown, T.A., Barlow, D.H., Money, R., Sholomskas, D.E., Woods, S.W., Gorman, J.M., Papp, L.A., 1997. Multicenter collaborative panic disorder severity scale. The American journal of psychiatry 154, 1571-1575.

Smit, F., Cuijpers, P., Oostenbrink, J., Batelaan, N., de Graaf, R., Beekman, A., 2006. Costs of nine common mental disorders: implications for curative and preventive psychiatry. The journal of mental health policy and economics 9, 193-200.

Smit, F., Lokkerbol, J., Riper, H., Majo, M.C., Boon, B., Blankers, M., 2011. Modeling the cost-effectiveness of health care systems for alcohol use disorders: how implementation of eHealth interventions improves cost-effectiveness. Journal of medical Internet research 13, e56.

Spek, V., Cuijpers, P., Nyklicek, I., Riper, H., Keyzer, J., Pop, V., 2007. Internet-based cognitive behaviour therapy for symptoms of depression and anxiety: a meta-analysis. Psychological medicine 37, 319-328.

Tyrer, P., Cooper, S., Salkovskis, P., Tyrer, H., Crawford, M., Byford, S., Dupont, S., Finnis, S., Green, J., McLaren, E., Murphy, D., Reid, S., Smith, G., Wang, D., Warwick, H., Petkova, H., Barrett, B., 2014. Clinical and cost-effectiveness of cognitive behaviour therapy for health anxiety in medical patients: a multicentre randomised controlled trial. Lancet 383, 219-225.

Ware, J.E., Jr., 2000. SF-36 health survey update. Spine 25, 3130-3139.

Warmerdam, L., Smit, F., van Straten, A., Riper, H., Cuijpers, P., 2010. Cost-utility and cost-effectiveness of internet-based treatment for adults with depressive symptoms: randomized trial. Journal of medical Internet research 12, e53.

1. https://www.nictiz.nl/module/360/914/Infographic%2520Medische%2520apps,%2520is%2520certificeren%2520nodig.pdf+&cd=1&hl=en&ct=clnk&gl=nl [↑](#footnote-ref-1)
