# Supplementary material for: Acceptability, effectiveness and cost-effectiveness of blended cognitive-behavioural therapy (bCBT) versus face-to-face CBT (ftfCBT) for anxiety disorders in specialised mental health care: A 15-week randomised controlled trial with 1-year follow-up
Source: PLoS One. 2021 Nov 12;16(11):e0259493. doi: 10.1371/journal.pone.0259493 (PMC8589191; doi:10.1371/journal.pone.0259493)
Supplement: S4 Appendix — (DOCX) [file pone.0259493.s004.docx]

# Appendix S4. Overview of measures administered at each assessment interval including mode of assessment

| **Assessment point** | **Assessments** | **Mode of administration** |
| --- | --- | --- |
| Baseline | SCID-I or MINI-Plus  BAI, BDI, BSI, Mastery Scale, WSAS, EQ-5D-5L, Tic-P | Ftf or telephone interview*  Self-administered |
| Week 7 (mid-treatment) | WAI-SR patient version, WAI-SR therapist version | Self-administered |
| Week 15 (post-treatment) | BAI, BDI, BSI, Mastery Scale, WSAS, EQ-5D-5L, Tic-P, CSQ-8, SUS | Self-administered |
| Week 52 (1-year follow-up) | BAI, BDI, BSI, Mastery Scale, WSAS, EQ-5D-5L, Tic-P | Self-administered |

_* Based on patient preference_
