# Supplementary material for: Acceptability, effectiveness and cost-effectiveness of blended cognitive-behavioural therapy (bCBT) versus face-to-face CBT (ftfCBT) for anxiety disorders in specialised mental health care: A 15-week randomised controlled trial with 1-year follow-up
Source: PLoS One. 2021 Nov 12;16(11):e0259493. doi: 10.1371/journal.pone.0259493 (PMC8589191; doi:10.1371/journal.pone.0259493)
Supplement: S5 Appendix — (DOCX) [file pone.0259493.s005.docx]

# Appendix S5. Unit costs used in the cost-effectiveness analysis

| **Description** | **Cost per visit** |
| --- | --- |
| **Direct medical costs**  Visits to specialized mental healthcare centre  Visit to independent psychologist  Online sessions  GP visits  Social worker visits  Physiotherapist visits  Visits to alternative healers  Visits to addiction services  Visits to self-help groups  Visits to company doctors | € 115.00  € 96.00  € 57.50  € 34.00  € 67.00  € 34.00  € 34.00  € 96.00  € 16.38  € 74.80 |
| **Direct non-medical costs** | € 0.19 per km travel  € 3.00 parking costs |
| **Productivity costs**  Short absence from work  Long absence from work  Presenteeism  Productivity loss in unpaid work | € 35.55 per h  € 35.55 per h  € 35.55 per h  € 14.32 per h |

_All unit costs were derived from the most recent Dutch guideline for economic evaluations. (Hakkaart-van Roijen et al., 2015)_
