# Supplementary material for: Acceptability, effectiveness and cost-effectiveness of blended cognitive-behavioural therapy (bCBT) versus face-to-face CBT (ftfCBT) for anxiety disorders in specialised mental health care: A 15-week randomised controlled trial with 1-year follow-up
Source: PLoS One. 2021 Nov 12;16(11):e0259493. doi: 10.1371/journal.pone.0259493 (PMC8589191; doi:10.1371/journal.pone.0259493)
Supplement: S6 Appendix — (DOCX) [file pone.0259493.s006.docx]

# Appendix S6. Total costs based on observed means and standard deviations

| **Costs** |  | **Blended CBT**  **Post-treatment** |  | **Face-to-face CBT**  **Post-treatment** |  | **Blended CBT**  **1-year follow-up** |  | **Face-to-face CBT**  **1-year follow-up** |
| --- | --- | --- | --- | --- | --- | --- | --- | --- |
|  | ***n*** | **Mean (SD)** | ***n*** | **Mean (SD)** | ***n*** | **Mean (SD)** | ***n*** | **Mean (SD)** |
| **Direct medical costs**  Visits to psychologist or psychiatrist  Online sessions  GP visits  Social worker visits  Physiotherapist visits  Visits to alternative healers  Visits to addiction services  Visits to self-help groups  Visits to company doctors | 34  34  34  34  33  34  34  34  34 | **€1358**  €815 (€645)  €331 (€162)  €32 (€38)  €39 (€140)  €75 (€205)  €14 (€38)  €0 (€0)  €0 (€0)  €52 (€92 | 43  43  43  43  43  43  43  43  43 | **€1588**  €1369 (€1457)  €7 (€44)  €58 (€56)  €34 (€141)  €29 (€87)  €28 (€140)  €9 (€59)  €6 (€31)  €48 (€84) | 32  32  32  32  32  32  32  32  32 | **€2343**  €1649 (€2942)  €111(€395)  €144 (€193)  €161 (€180)  €190 (€372)  €3 (€19)  €0 (€0)  €6 (€25)  €79 (€180) | 39  39  40  40  40  40  39  39  40 | **€2290**  €1836 (€3662)  €50 (€240)  €105 (€134)  €21 (€102)  €160 (€315)  €16 (€99)  €15 (€66)  €28 (€125)  €59 (€146) |
| **Travel costs** | 33 | **€28 (€40)** | 43 | **€59 (€64)** | 32 | **€42 (€79)** | 39 | **€87** (€233) |
| **Productivity costs**  Short absence from work  Long absence from work  Presenteeism  Productivity loss in unpaid work | 34  34  34  34 | **€3129**  €491 (€1804)  €0 (€0)  €1432 (€3000)  €1206 (€4061) | 43  43  43  43 | **€2364**  €729 (€2886)  €176 (€894)  €1055 (€3112)  €404 (€898) | 33  32  33  31 | **€4493**  €1395 (€6399)  €0 (€0)  €2006 (€6089)  €1092 (€2646) | 39  39  39  39 | **€4397**  €864 (€2204)  €183 (€1010)  €1907 (€3714)  €1443 (€3289) |
| **Total** |  | €**4515** |  | €**4011** |  | **€6878** |  | **€6774** |
